# *AGP* and *EXO‐LIKE* genes promote brassinosteroid‐dependent anisotropic growth

**DOI:** 10.1111/nph.71063

**Published:** 2026-03-08

**Authors:** Daria Novikova, Surbhi Rana, Kunkun Li, H. Nicholay Diaz‐Ardila, Nicola Trozzi, Luis Alonso Baez, Thorsten Hamann, Mateusz Majda, Christian S. Hardtke

**Affiliations:** ^1^ Department of Plant Molecular Biology University of Lausanne Lausanne CH‐1015 Switzerland; ^2^ Institute for Biology, Faculty of Natural Sciences Norwegian University of Science and Technology Trondheim 7491 Norway

**Keywords:** arabinogalactan peptides, arabinogalactan proteins, cellular anisotropy, EXORDIUM, organ stiffness, root

## Abstract

- The brassinosteroid pathway promotes anisotropic cell expansion; however, the effectors in this process remain unclear. Candidates include *ARABINOGALACTAN PROTEIN* (*AGP*) genes, which are prominent brassinosteroid‐responsive transcriptional targets, and *EXORDIUM (EXO)‐LIKE* (*EXL*) genes. Here, we examined whether *AGP* and *EXO/EXL* genes mediate anisotropic cell expansion.
- AGPs are evolutionarily ancient glycoproteins, which are secreted and attached to the plasma membrane. Plant genomes contain dozens of potentially redundant *AGP* genes. Their roles have mostly been inferred from the phenotypes of beta‐glycosyltransferase mutants with impaired AGP glycosylation. Compared with AGPs, little is known about the smaller family of secreted EXO/EXL proteins.
- Here, we investigated brassinosteroid‐dependent *AGP* and *EXO/EXL* gene expression patterns in Arabidopsis roots and created loss‐of‐function mutants for preponderant genes. Whereas single, double and triple *agp* mutants appeared wild‐type, combinatorial quadruple to sextuple *agp2/4/10/11/13/22/24* mutants displayed longer, frequently aberrant root hairs and shorter yet thicker roots. These traits reflect an underlying reduced cellular anisotropy associated with increased organ stiffness, which were also observed in hypocotyls. Combinatorial *exo/exl1/2/3/4/5/6/7* sextuple or septuple mutants displayed a qualitatively similar but quantitatively weaker phenotype than combinatorial *agp* mutants.
- Collectively, our data suggest that classical/peptide AGPs and EXO/EXLs promote brassinosteroid‐dependent anisotropic growth to different extent.

## Introduction

Brassinosteroids are key growth regulators that signal through a receptor kinase pathway, which evolved in angiosperms (Kim & Russinova, 2020). In Arabidopsis (*Arabidopsis thaliana*), the major active brassinosteroid is brassinolide, which triggers a phospho‐transfer cascade upon binding to and stimulating the activity of the BRASSINOSTEROID‐INSENSITIVE 1 (BRI1) receptor kinase. The signal is eventually transduced to redundant transcription factors (Kim *et al*., 2009), chiefly BRASSINAZOLE RESISTANT 1 (BZR1) and BRI1 EMS SUPPRESSOR 1 (BES1) (a.k.a. BZR2), which then up‐ or downregulate target genes depending on context and cofactors (Kim *et al*., 2009; Sun *et al*., 2010; Yu *et al*., 2011). One of the dominant outputs of brassinosteroid signaling in the root is the stimulation of growth through the promotion of cellular anisotropy (Fridman *et al*., 2021; Graeff *et al*., 2021). It remains unclear however which among the hundreds of brassinosteroid‐regulated target genes are the downstream effectors in this process. Because brassinosteroids impact cell wall composition and structure (Rao & Dixon, 2017; Percio *et al*., 2024), candidates include genes encoding ARABINOGALACTAN PROTEINs (AGPs), which are coherently positively regulated by the brassinosteroid signaling pathway (Clark *et al*., 2021; Nolan *et al*., 2023) and correlate with root cell elongation in Arabidopsis as well as in the monocotyledon model, Brachypodium (*Brachypodium distachyon*) (Pacheco‐Villalobos *et al*., 2016; Graeff *et al*., 2021). Likewise, genes of the *EXORDIUM* (*EXO*)‐*LIKE* (*EXL*) family respond to some degree to brassinosteroids (Clark *et al*., 2021; Graeff *et al*., 2021; Nolan *et al*., 2023) and were suggested to play a role in cell expansion (Schroder *et al*., 2009, 2012). Here, we sought to determine whether *AGP* and *EXO/EXL* genes play a role in anisotropic cellular growth.

AGPs are a diverse family of glycoproteins that are found throughout the green lineage as well as in brown algae (Seifert & Roberts, 2007; Herve *et al*., 2016; Ma & Johnson, 2023). They are characterized by a protein backbone rich in hydroxyprolines, which are O‐linked to arabinogalactan glycan chains that can constitute up to 90% of an AGP's mass. Their protein sequences harbor clusters of proline, alanine, serine and threonine (PAST), which can be combined with other domains in the so‐called chimeric AGPs (Seifert & Roberts, 2007; Silva *et al*., 2020; Ma & Johnson, 2023). However, classical AGPs only comprise the AGP hallmark features of an N‐terminal signal peptide for delivery to the cell surface, a PAST‐rich sequence of 100–150 amino acids, and a hydrophobic region at the C terminus that directs addition of a glycosylphosphatidylinositol lipid anchor for attachment to the extracellular face of the plasma membrane (Schultz *et al*., 2000; Muniz & Riezman, 2016; Silva *et al*., 2020). Finally, the arabinogalactan peptides are overall similar to classical AGPs but have protein backbones as short as 10 amino acids (Sherrier *et al*., 1999; Schultz *et al*., 2000; Seifert & Roberts, 2007). AGPs can be stained and coarsely quantified with the beta‐Yariv reagent, which precipitates AGPs and thereby also interferes with their function (Yariv *et al*., 1967; Nothnagel, 1997; Prerovska *et al*., 2021).

Plant genomes contain dozens (100–200) of AGP‐encoding genes, and even for the classical and peptide subclasses typically 10–20 each can be found (Seifert & Roberts, 2007; Ma & Zhao, 2010; Bartels & Classen, 2017; Ma *et al*., 2017; Mueller *et al*., 2023), pointing to potential redundancy. Indeed, single knockout mutants for *AGP* genes identified so far in Arabidopsis have no or comparatively mild phenotypes. For example, the role of *AGP6* in pollen tube maintenance is only evident in homozygous *agp6* mutants that are combined with *agp11* mutation in heterozygous state (Coimbra *et al*., 2009). Reported single mutant phenotypes include *agp4*, in which pistils show impaired pollen tube blockage (Pereira *et al*., 2016), and *agp15* and *agp21*, which both display altered root hair patterning (Borassi *et al*., 2020). Also, knockout of the Physcomitrium (*Physcomitrium patens*) homolog of Arabidopsis *AGP1* results in somewhat reduced cell elongation (Lee *et al*., 2005). The functions of the vast majority of individual AGPs thus have not been clearly defined yet.

Compared with AGPs, little is known about *EXO/EXL* genes. The founding member, *EXO*, was originally described as a brassinosteroid effector in leaf cell expansion (Schroder *et al*., 2009). In Arabidopsis, *EXO/EXLs* constitute a small family of eight genes (Schroder *et al*., 2012), which encode proteins of unknown biochemical function that contain a signal peptide and are secreted into the apoplast (Schroder *et al*., 2009). Similar to AGPs, they appear to be evolutionarily ancient because homologs can be found in bacterial genomes (Schroder *et al*., 2009). The effect of *EXO* loss‐of‐function remains unclear. On the one hand, a relatively strong growth retardation phenotype has been described for a T‐DNA insertion mutant (*exo‐1*), which also implicated *EXO* in the modification of brassinosteroid responses (Schroder *et al*., 2009). On the other hand, a promoter trap line of *EXO* with substantially reduced gene expression as well as *EXO* antisense plants did not display any noticeable phenotype (Farrar *et al*., 2003).

## Materials and Methods

### Plant materials and growth conditions

Seeds were surface‐sterilized using 70% ethanol, sown onto half‐strength Murashige and Skoog (½ MS) agar medium (1% agarose) supplemented with 0.3% sucrose and stratified for 2 d at 4°C. Plants were grown under continuous white light (intensity *c*. 120μE) at 22°C under a 16 h : 8 h, light : dark photoperiod. All mutants and marker lines were in the *Arabidopsis thaliana L*. Columbia‐0 (Col‐0) wild‐type (WT) background. The *exo‐1* mutant was obtained from the Nottingham Arabidopsis stock center. To study root hair phenotypes, plants were grown on minimal medi (Wymer *et al*., 1997). To study hypocotyls, *exo/exl* mutants were grown on ½ MS without sugar. After stratification, plates intended for hypocotyl studies were exposed to white light for up to 24 h to induce uniform germination and then transferred to complete darkness. Seedlings were grown vertically in a growth chamber at 22°C under dark conditions to allow hypocotyl elongation.

### Generation of transgenic lines, *agp* and exo/exl mutants

For transcriptional reporters, the promoter regions were PCR‐amplified from Col‐0 genomic DNA and cloned into the pCAMBIA1305.1‐NLS‐3xCITRINE plasmid using the NEBuilder® HiFi DNA Assembly Cloning Kit (New England Biolabs, Ipswich, MA, USA) for *agp* mutants and the Gateway® system for *exo/exl* mutants. All constructs were transformed by heat shock into the *Agrobacterium tumefaciens* GV3101 pMP90 strain and then transformed into plants by the floral dip method. At least five independent transgenic lines were analyzed for expression patterns, and one line showing a representative signal and normal segregation was selected for further studies. To generate CRISPR/Cas9 lines, spacers designed for SpCas9‐targeting sgRNAs were cloned using the oligo annealing technique and then ligated into BbsI‐linearized Gateway‐entry plasmids. For multiplex targeting of genes, the two sgRNAs utilized were cloned into two vectors, and these vectors were cotransformed together into the Col‐0 accession. Dataset S3 contains details about the primers used in cloning and multiple CRISPR/Cas9 lines generation.

### Quantitative real‐time PCR

Whole roots were collected from 10‐d‐old seedlings for total RNA extraction (Direct‐zol RNA Miniprep Kit; ZYMO RESEARCH, Irvine, CA, USA), and cDNAs were produced by reverse transcriptase (Invitrogen, Carlsbad, CA, USA). Quantitative real‐time PCR (qPCR) was performed on three technical and biological replicates using MESA Blue qPCR MasterMix Plus for SYBR assay Low Rox (Eurogentec, Seraing, Belgium).

### Treatment assays

To analyze the effects of the beta‐glucosyl Yariv reagent (Biosupplies Ltd, Yagoona, NSW, Australia), treatments were conducted at concentrations of 400 nM and 5 μM, and alpha‐galactosyl Yariv reagent (Biosupplies Ltd) was used as a control. Postgermination treatment: 4‐d‐old seedlings grown on ½ MS medium were transferred to ½ MS medium supplemented with the appropriate concentration of alpha‐Yariv or beta‐Yariv. Direct germination treatment: Seeds were directly sown and germinated on ½ MS plates containing the appropriate concentration of alpha‐Yariv or beta‐Yariv reagent. Seedlings subjected to these treatments were grown under the same growth conditions as described previously. Seedlings treated with Yariv reagents were imaged at two developmental stages: (1) 8‐d‐old seedlings transferred to Yariv‐containing media were imaged after 4 d of treatment, and (2) 15‐d‐old seedlings that were germinated and grown directly on Yariv‐containing media. To analyze brassinolide effects on *agp* and *exo/exl* mutants, we transferred 3‐d‐old seedlings from ½ MS medium on medium supplemented with 50 pM brassinolide (Sigma‐Aldrich) and measured their root length 5 d thereafter. In another set of experiments, mutants were grown on ½ MS medium supplemented with 1 μM brassinazole (Tokyo Chemical Industry, Tokyo, Japan) for 3 d, then transferred on medium supplemented with 100 pM brassinolide, and measured after 5 and 7 more days. Images of the seedlings were captured using a flatbed scanner, and roots were traced in the ImageJ software with the Simple Neurite Tracer Plugin.

### Statistical analyses and reproducibility

The data were subjected to statistical analysis using GraphPad Prism v.10.4.0. Robust regression and Outlier removal (ROUT) was performed to detect (rare) outliers, which were subsequently removed. Statistical tests applied are indicated in the figure legends and were always two‐sided. All experiments were performed at least twice. Differences between treatments and control were assessed using one‐way ANOVA followed by Tukey's *post hoc* test. An adjusted *P*‐value < 0.05 was considered statistically significant.

### Tissue fixation, staining and microscopy

For confocal microscopy, the roots of 3‐ to 10‐d‐old seedlings were fixed in 4% paraformaldehyde (PFA) in phosphate buffered saline (PBS) buffer and washed in PBS. For clearing of the samples, seedlings were transferred into ClearSee solution (Kurihara *et al*., 2015) for 7 d at 4°C with regular changes of the clearing solution. Cleared roots were stained with 0.25 mg ml^−1^ Calcofluor White (CCFW; Sigma, product no. F3543) and washed with ClearSee solution. Samples were imaged on a Stellaris Leica confocal microscope using a 405‐nm laser for CCFW excitation and recording of the cell wall signal in the 450–480 nm range. The seedlings stained with COS488 probe (Mravec *et al*., 2017) at a 1 : 500 dilution in ddH_2_O were incubated for 15 min, then washed with water, mounted on a glass slide and covered with a coverslip. Confocal microscopy was performed on a Leica Stellaris confocal microscope. Reporter lines were imaged live with propidium iodide (PI) cell wall counterstaining.

To study root hair phenotype and Yariv staining, a light microscope (Zeiss AxioZoom) was used. Scanning electron microscopy (SEM) was performed using a FEI Quanta FEG 250 equipped with SE, BSE, STEM and E‐SEM detectors. Low vacuum and ESEM capability enabled charge‐free imaging and analysis of nonconductive and/or hydrated specimens. For transmission electron microscopy (TEM), mature parts of the roots (5 mm above the tip) were resin‐embedded, sectioned and imaged on a TEM JEOL 2100Plus instrument. Two cross sections per genotype were imaged. For cell wall width quantification in TEM images, three measurements were taken for each outer epidermal cell wall and for the cortex–epidermis cell wall junctions.

For root width, cell length and cell width measurements, Z‐stacks were used. Cell length and width were measured once per cell, while root widths were measured three times per root ortho‐projected images. To quantify COS488 intensity differences, Z‐stacks containing 100–113 optical sections with a fixed step size were analyzed. Maximum projection images were generated, and the mean intensity was measured using a fixed region of interest (ROI), with three measurements taken per root.

### Stiffness measurements

A microextensometer setup was used as previously described (Majda *et al*., 2022). The equipment and lights were switched on several hours before the experiment to stabilize thermal conditions. The seedlings were isolated and attached to pinched laboratory tags (Tough‐Tags™; Diversified Biotech, Dedham, MA, USA). Next, the samples were left in a water bath to stabilize their osmotic conditions, as it could influence turgor pressure and cause cell deformation. The samples were attached to the microextensometer arms, and the MorphoRobotX process was used to move the sensor against the immobilized arm, thereby acquiring force–displacement curves. The sample stiffness was calculated as the force per unit width divided by the percentage of displacement.

### Brillouin microscopy

Brillouin microscopy was performed using a custom‐built confocal Brillouin microscope based on a two‐stage imaged phase array, VIPA (Zhang & Scarcelli, 2021). Excitation was performed using a 532‐nm laser (Cobolt; Hübner Photonics, Solna, Sweden). The laser was guided to the back port of an inverted Leica SP8 confocal microscope. Samples were illuminated using a 40× water immersion objective NA 1.1 (Leica, Wetzlar, Germany), and the scattered light was collected using a backscattered geometry. The spectrometer included two‐stage VIPA etalons (OP‐6721‐3371‐2, 500–600 nm, 30 GHz free spectral range – Light Machinery) and a Lyot stop (Edri *et al*., 2017). An ORCA‐Quest qCMOS camera (C15550‐20UP; Hamamatsu Photonics, Shizuoka, Japan) was used to record the Brillouin spectra. Images were acquired using a physical pinhole of 1 Airy unit, 100‐ms acquisition time, 1 μm stage step sizes and 10–12 mW laser power. The microscopy room was maintained at 20°C, and the laser was mounted on a heated plate set to 37°C (according to manufacturer recommendations). To check for perturbations and phototoxicity, samples were inspected by transmitted‐light wide‐field illumination before and after the Brillouin spectra was recorded. No visible differences were detected. For each pixel, the spectrum was recorded four times and summed. Water and methanol were used as reference samples to calibrate the frequency axis. During Brillouin spectra recording, the confocal microscope stage movement and image acquisition were controlled using the HCImage software from Hamamatsu. A custom‐made Matlab script was used to extract the positions of the Brillouin peaks from the recorded images after fitting them with a Lorentzian function. Peak positions, along with the calibration samples, were used to calculate the frequency shift (i.e. the frequency difference between the Stokes/anti‐Stokes peaks and the Rayleigh peak). The frequency shift is used as a proxy for the elastic behavior of a material (i.e. its longitudinal modulus), as described by the formula:

$$v_{B}=\frac{2n}{\lambda }\sqrt{\frac{M^{\prime }}{\rho }}\sin\frac{\theta }{2}$$

where ν_B_ is the frequency shift, n the refractive index, λ the excitation laser frequency, M′ the real part of the longitudinal modulus, ρ the material density and θ the scattering collection angle. Brillouin values are reported as the relative frequency shift of the sample as compared to water.

### Bioinformatics

Single‐cell RNA sequencing (scRNA‐seq) expression data were obtained from the publicly available dataset published by Nolan *et al*., 2023. The dataset encompasses transcriptional profiles across various conditions and treatments in *A. thaliana*. For this study, only the no‐treatment condition and treatment samples were used. To analyze the expression patterns of the *AGP* and *EXO/EXL* gene families, raw read counts for all genes of interest were aggregated from the scRNA‐seq dataset for each cell or sample, the aggregated expression data were normalized using the counts per million method to adjust for sequencing depth, and a logarithmic transformation (log_2_(*x* + 1)) was applied to reduce variability and highlight expression trends. For the treatment samples, aggregated reads were processed without considering tissue‐specific classifications, allowing for a comprehensive view of expression under treatment conditions. Heatmaps of the normalized and log‐transformed expression data were generated using Python. The analysis utilized libraries such as Pandas for data manipulation, Seaborn and Matplotlib for visualization, and NumPy for numerical operations. Heatmaps were constructed to depict the relative expression of *AGP* genes across no‐treatment and treatment conditions. Statistical analyses, including comparison of expression levels between conditions, were performed where applicable. Differences were assessed using appropriate statistical methods (e.g. *t*‐tests or nonparametric tests) implemented in Python with libraries such as SciPy or Statsmodels. A 116 amino acid sequences aligned with Clustal Omega (https://www.ebi.ac.uk/jdispatcher/msa/clustalo) using default parameters were used to build phylogenetic trees with Iq‐Tree (http://www.iqtree.org) using standard parameters. The ITOLv7 (https://itol.embl.de) software was used for tree visualization.

## Results

### Brassinosteroid‐induced *AGP* genes redundantly limit root growth

**Fig. 1:**
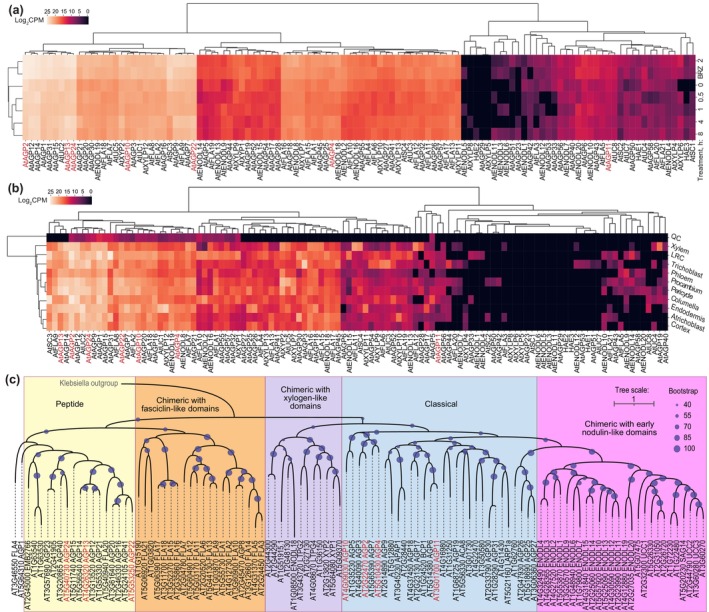
Classical and peptide *ARABINOGALACTAN PROTEIN* (*AGP*) genes are brassinosteroid‐responsive. (a) Heatmap of Arabidopsis *AGP* gene expression (single‐cell mRNA sequencing) upon brassinolide treatment of roots depleted of brassinosteroids by prior brassinazole treatment (data from Nolan *et al*., 2023). (b) Heatmap of tissue‐specific Arabidopsis  gene expression in roots (data from Nolan *et al*., 2023). (c) Phylogenetic tree of Arabidopsis AGPs with the different subclasses shaded in different colors. Note the outlier positions of FLA4 (chimeric with fasciclin‐like domain) and AGP1 (classic). Genes for which mutants were obtained are highlighted in red in (a–c).

**Fig. 2:**
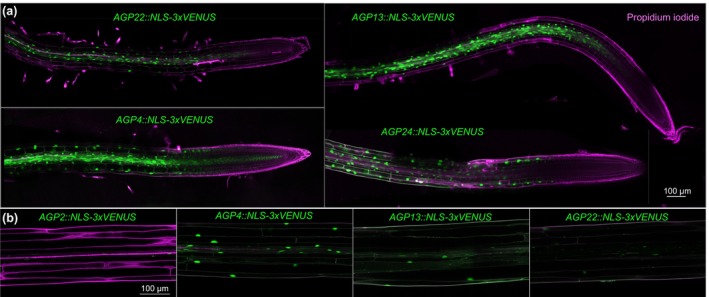
Expression patterns of selected *AGP* genes. (a, b) Confocal microscopy of root tips (a) and hypocotyls (b) expressing transgenic transcriptional reporters (nuclear localized VENUS protein, green fluorescence) with promoters of the indicated *AGP* genes in WT (Col‐0) background (cells counterstained with propidium iodide, magenta fluorescence). Note the general increase in expression as cells start to expand in roots.

**Fig. 3:**
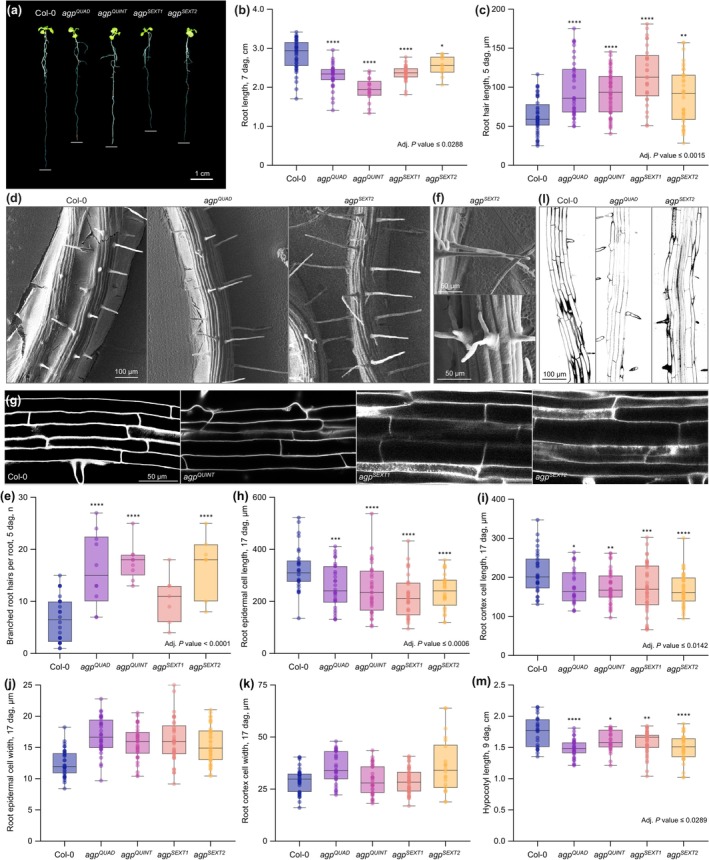
Root phenotypes of *agp* multiple mutants. (a) Representative images of 10‐d‐old (10 dag) seedlings of the indicated genotypes. Root tip positions are marked by white bars. (b) Root length measurements. (c) Root hair length measurements. (d) Scanning electron microscopy (SEM) images of roots. Note the longer root hairs in the mutants. (e) Quantification of the frequency of branched root hairs. (f) SEM images of branched root hairs in *agp* ^*SEXT2*^ mutants. (g) Close‐up confocal microscopy images of epidermal cells in indicated genotypes. (h–k) Quantification of epidermal (h, j) and cortex (i, k) cell length (h, i) and width (j, k). (l) Confocal microscopy images of representative roots (7 dag). Note that mutant roots appear to be slightly thicker than WT. (m) Hypocotyl length measurements (7 dag, dark‐grown). Statistically significant differences (asterisks) compared to Col‐0 were determined by ordinary one‐way ANOVA followed by Tukey's multiple comparison test, two‐sided. *agp* ^*QUAD*^: *agp4/13/22/24* quadruple mutant; *agp* ^*QUINT*^: *agp4/11/13/22/24* quintuple mutant; *agp* ^*SEXT1*^: *agp4/10/11/13/22/24* sextuple mutant; *agp* ^*SEXT2*^: *agp2/4/11/13/22/24* sextuple mutant; seedlings were grown on ½ strength MS media supplemented with 0.3% sucrose, except for the root hair assays in (c) and (e), which were performed on minimal media. Box plots display 2nd and 3rd quartiles and the median, whiskers indicate maximum and minimum. *, *P* ≦ 0.05; **, *P* ≦ 0.01; ***, *P* ≦ 0.001; ****, *P* ≦ 0.0001.

We first analyzed the *AGP* gene family to select the most promising candidates based on brassinosteroid response, tissue‐specificity and absolute expression level. In a set of single‐cell mRNA sequencing (scRNAseq) data from roots pretreated with the brassinosteroid biosynthesis inhibitor brassinazole and subsequently exposed to brassinolide (Nolan *et al*., 2023), we identified 112 of the 116 Arabidopsis *AGP* genes expressed in the different root tissues (Fig. 1a; Supporting Information Dataset S1a–c). Among them, 46 were reportedly significantly induced by brassinolide treatment within 30 min to 8 h in an independent bulk RNAseq experiment (Clark *et al*., 2021) (Fig. S1b; Dataset S1d,e). These included classical and peptide *AGP* genes identified as sentinels of cellular anisotropy in brassinosteroid‐blind mutants (Graeff *et al*., 2021), which were also brassinosteroid‐responsive in another dataset (Chaiwanon & Wang, 2015). We concentrated our efforts on a subset of classical/peptide *AGP* genes that are collectively expressed at relatively high absolute levels across the different root tissues, although generally strongest in the vascular cylinder (Figs 1b,c, S2a; Dataset S1b), as independently confirmed for a subgroup by transcriptional reporter genes (Figs 2a, S2b). Both surveys also indicated that *AGP* genes are already expressed in dividing, meristematic cells but typically upregulated once cells enter the transition toward elongation and differentiation. Through CRISPR/Cas9‐mediated gene editing in the Columbia‐0 (Col‐0) reference accession, we could generate mutants in the classical *AGP* genes, *AGP2*, *AGP4*, *AGP10* and *AGP11*, as well as in the *AGP* peptide genes, *AGP13*, *AGP22* and *AGP24* (Fig. S3). These mutations can be considered loss‐of‐function since the introduced nucleotide insertions or deletions lead to frameshifts either within the AGP moiety of the protein sequence (*AGP2*, *AGP4*, *AGP10*, *AGP11*), the propeptide (*AGP13*, *AGP22*) or the signal peptide (*AGP24*). Generally, we did not observe any apparent quantitatively robust mesoscopic phenotypes in various single, double and triple mutant combinations we obtained. However, a visually obvious and statistically significant root growth phenotype was observed in *agp4/13/22/24* quadruple (*agp* ^*QUAD*^) mutants and further consolidated in *agp4/11/13/22/24* quintuple (*agp* ^*QUINT*^), *agp4/10/11/13/22/24* sextuple (*agp* ^*SEXT1*^) and *agp2/4/11/13/22/24* sextuple (*agp* ^*SEXT2*^) mutants, in that root growth vigor was reduced up to *c*. 35% (Fig. 3a,b). Consistent with a presumed AGP reduction, beta‐Yariv staining was reduced throughout the roots of the mutants (Fig. S4a,b).

### *AGP* genes redundantly promote cellular anisotropy in roots and hypocotyls

Light microscopy analyses revealed a pronounced root hair phenotype in all mutant combinations (Fig. S4c), which manifested in overall longer root hairs closer to the meristematic zone (Fig. 3c,d) and their frequent branching throughout the whole root (Fig. 3e) as further illustrated by SEM (Fig. 3d,f). Some branching can occur in WT plants, mainly close to the hypocotyl, but branching in the *agp* mutants was very pronounced starting from the first root hairs emerging near the meristem. Moreover, confocal microscopy of mature root sections indicated that generally, cells were shorter in all four mutants as exemplified by epidermal and cortex cells (Fig. 3g–i). Moreover, although not statistically significant, in tendency, cells were wider (Fig. 3j,k), consistent with thicker mutant roots (Figs 3l, S4d). Together, these data are consistent with an overall reduced cellular anisotropy in the mutants. Anisotropic cellular growth is also pertinent in other growth processes such as hypocotyl elongation, and prominent *AGP* expression has for example been observed in shade avoidance response (Kohnen *et al*., 2016). Consistently, we observed complementary expression of *AGP2*, *AGP4*, *AGP13* and *AGP22* throughout hypocotyls (Fig. 2b) and matching reduced cellular anisotropy in the *agp* mutants; that is, dark‐grown hypocotyls were significantly shorter than WT (up to *c*. 15%) in all four mutants (Fig. 3m) and composed of shorter cells (Fig. S4e,f). In summary, the morphological phenotypes of the *agp* multiple mutants investigated can be parsimoniously explained by a reduced cellular anisotropy that *a priori* results from impaired longitudinal cell expansion.

### *agp* loss‐of‐function mutants display stiffer roots and hypocotyls

**Fig. 4:**
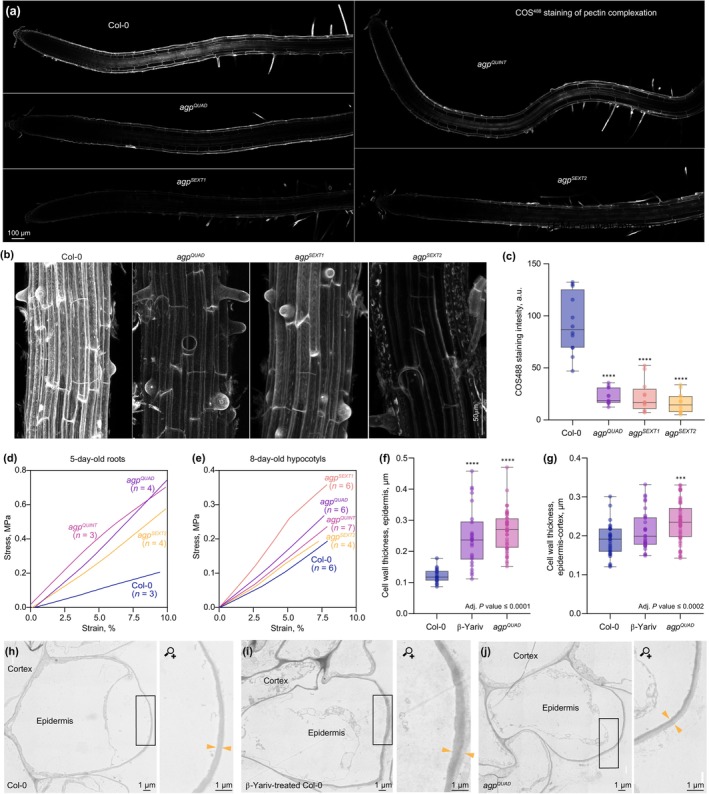
Cell wall and mechanical features of *agp* multiple mutants. (a) Representative confocal microscopy images of roots stained for pectin complexity using the COS488 probe. (b, c) Close‐ups of maximum projection 3D image stacks of COS488‐stained roots (b) and quantifications of maximum projection COS488 staining intensity (c). (d, e) Microextensometer force–displacement curves of roots (d) and dark‐grown hypocotyls (e), smoothed average, corrected for sample thickness. (f, g) Thickness measurements for outer epidermal cell walls (f) or epidermis–cortex junction cell walls (g) obtained from transmission electron microscopy (TEM) images. *n* = 18–44 (f) and 30–54 (g) cells from two different roots. Statistically significant differences (asterisks) compared to Col‐0 were determined by ordinary one‐way ANOVA followed by Tukey's multiple comparison test, two‐sided. (h–j) TEM images of representative cells from Col‐0 wild‐type (h), Col‐0 treated with beta‐Yariv agent (i), and *agp* ^*QUAD*^ mutants, with boxed areas magnified. Arrowheads point out the outer cell wall of epidermal cells. Box plots display 2^nd^ and 3^rd^ quartiles and the median, whiskers indicate maximum and minimum. ***, *P* ≦ 0.001; ****, *P* ≦ 0.0001.

Manipulation of cell wall composition often impacts mechanical cell wall properties, which is thought to play a key role in cellular morphogenesis (Peaucelle *et al*., 2011; Jonsson *et al*., 2022). Both arabinans and galactans have been found to be associated with pectins (Verhertbruggen *et al*., 2013; Takahashi *et al*., 2024), which constitute a major fraction of the cell wall components, and indeed AGPs that are covalently linked to pectin were recently reported to represent a major form of AGPs (Tan *et al*., 2024). Staining of roots with the fluorescent chitosan oligosaccharide (COS) probe COS488, which binds to the homogalacturonan portion of pectic polysaccharides and its derivative oligogalacturonate fragments (Mravec *et al*., 2014), revealed a strongly reduced signal in the mutant roots (Fig. 4a–c). This observation suggests that the pectin structure was altered in the cell walls of the *agp* mutants (Mravec *et al*., 2014, 2017). Because the extent of cross‐linked pectin is an important determinant of cell wall mechanics (Peaucelle *et al*., 2011; Shaw, 2019; Senechal *et al*., 2024), we investigated whether mechanical features are affected in roots of the *agp* mutants. We first employed Brillouin microscopy to measure the longitudinal modulus and thereby assess the viscoelastic properties of cell walls *in vivo* and noninvasively with single‐cell resolution (Shaw, 2019; Bacete *et al*., 2022; Alonso Baez & Bacete, 2023). These experiments did not detect statistically significant differences in the walls of epidermal and ground tissue cells (Fig. S5a), while the walls of the smaller pericycle and vascular cells could not be measured due to technical limitations. To assess whether we could nevertheless detect an organ‐scale change that may emerge from effects in the vasculature, from accumulated small effects along cell files or from the altered cell geometry, we employed a microextensometer setup (Majda *et al*., 2022; Trozzi *et al*., 2025) that measures Young's modulus to obtain strain–stress curves from extension of 5‐d‐old roots along their longitudinal axes. These experiments revealed that substantially more force had to be applied for the initial elastic extension of *agp* mutant roots as compared to WT (Fig. 4d). Consistent with this observation, similar results were obtained with 8‐d‐old dark‐grown hypocotyls (Fig. 4e). For hypocotyls, we could further verify the phenotype by measuring creep compliance (Park & Cosgrove, 2012; Trozzi *et al*., 2025), which was significantly reduced in the *agp* ^*QUAD*^ mutant as compared to WT (Fig. S5b). This implies that the cell walls were stiffer upon *AGP* knockout because of their inability to respond as efficiently to constant mechanical stress as the WT. Moreover, TEM images of root cross sections (Fig. 4h–j) indicated an increase in cell wall thickness in the *agp* ^*QUAD*^ mutants, which was also observed in beta‐Yariv‐treated WT roots (Fig. 4f,g). Quantitatively, thickening of the cell wall was more prominent in the beta‐Yariv‐treated WT roots, presumably because it interfered with the function of most AGPs, yet it indicates that knockout of a few brassinostroid‐regulated *AGPs* yields a qualitatively similar phenotypic change. In summary, the *agp* mutants displayed an organ‐scale mechanical phenotype of collectively stiffer roots and hypocotyls that is associated with reduced cellular anisotropy.

### *EXO/EXL* genes display variable brassinosteroid response

**Fig. 5:**
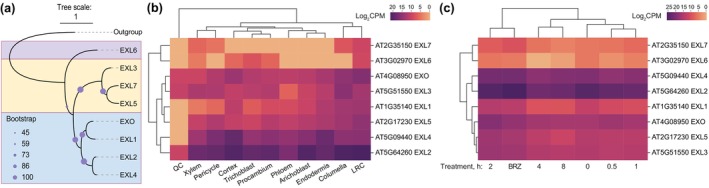
Phylogeny and expression of *EXORDIUM (EXO)‐LIKE* (*EXL*) family genes. (a) Phylogenetic tree of Arabidopsis *EXO/EXL* genes with the unequivocally separate clades shaded in different colors. (b) Heatmap of tissue‐specific Arabidopsis *EXO/EXL* gene expression in roots (data from Nolan *et al*., 2023). (c) Heatmap of Arabidopsis *EXO/EXL* gene expression (single‐cell mRNA sequencing) upon brassinolide treatment of roots depleted of brassinosteroids by prior brassinazole treatment (data from Nolan *et al*., 2023).

Compared with *AGP* genes, selection of pertinent *EXO/EXL* genes was more straightforward due to the much smaller complexity of the gene family (Fig. 5a). In terms of expression, most *EXO/EXL* genes were expressed to variable degrees across root tissues, with *EXO*, *EXL2*, *EXL3* and *EXL4* emerging as dominant but spatially distinct (Fig. 5b; Dataset S2a,b), while brassinosteroid response was relatively mild or even absent in scRNAseq data (Nolan *et al*., 2023) (Fig. 5c; Dataset S2c) as compared to *AGPs*. Moreover, whereas *AGP* genes responded overwhelmingly positively to brassinosteroid stimulus (Dataset S1e), *EXO/EXL* response was more varied in that some genes were consistently upregulated (*EXO*, *EXL1*, *EXL3*), while others were consistently downregulated (*EXL2*, *EXL4*) in bulk RNAseq (Dataset S2d,e).

### Loss of *EXO* function does not result in conspicuous growth phenotypes

In our initial experiments, we revisited the previously described *exo‐1* mutant that carries a homozygous T‐DNA insertion in the *EXO* coding region (Schroder *et al*., 2009). However, we did not observe the previously reported shoot growth phenotype (Schroder *et al*., 2009), and consistently, we also observed neither the reported root growth phenotype (Fig. S6a,b), nor a hypocotyl phenotype (Fig. S6c), nor the reported hypersensitive response to brassinosteroid treatment (Fig. S6d,e). The absence of any conspicuous phenotype was confirmed with an independent *exo* allele (*exo‐2*, a single nucleotide insertion leading to a frameshift; Fig. S7) that we created by CRISPR/Cas9‐mediated gene editing in Col‐0. Our data are thus in line with another report that did not detect any phenotypes upon *EXO* downregulation (Farrar *et al*., 2003).

### *EXO/EXL* genes display differential expression patterns in roots and hypocotyls

**Fig. 6:**
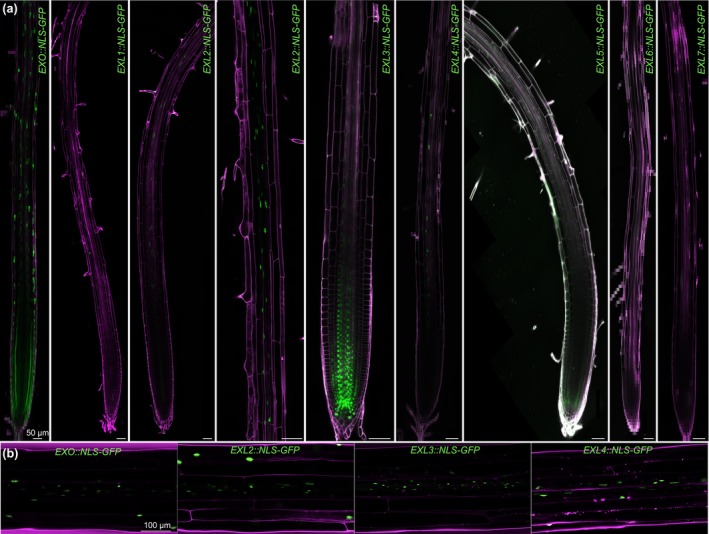
Expression patterns of *EXO/* *EXL* genes. (a, b) Confocal microscopy of root tips (a) and hypocotyls (b) expressing transgenic transcriptional reporters (nuclear localized GFP protein, green fluorescence) with promoters of the indicated *EXO/EXL* genes in wild‐type (Col‐0) background (cells counterstained with propidium iodide, magenta fluorescence).

To experimentally monitor expression levels and profiles, we created transcriptional reporters for all *EXO/EXL* gene family members. Their analysis in roots indicated spatially differential yet overlapping gene expression in meristematic, elongating as well as mature root cells (Fig. 6a) with a dominant expression of *EXO* throughout the root, *EXL2* in mature regions and *EXL3* in the meristem. In terms of expression range and levels, *EXO*, *EXL2* and *EXL3* were also most prominent, whereas *EXL6* and *EXL7* were barely detectable in line with RNAseq data (Dataset S2b). Overall, these observations fit the expression profiles obtained from scRNAseq data, some discrepancy notwithstanding (Fig. S8). In hypocotyls, only *EXO*, *EXL2*, *EXL3* and *EXL4* could be detected, with the former two apparently expressed throughout all tissues and the latter two more prominently in the stele (Fig. 6b). In summary, our reporters indicate that *EXO/EXL* genes are expressed in overlapping as well as distinct patterns throughout the root and hypocotyl.

### *EXO/EXL* genes redundantly promote anisotropic root and hypocotyl cell elongation

**Fig. 7:**
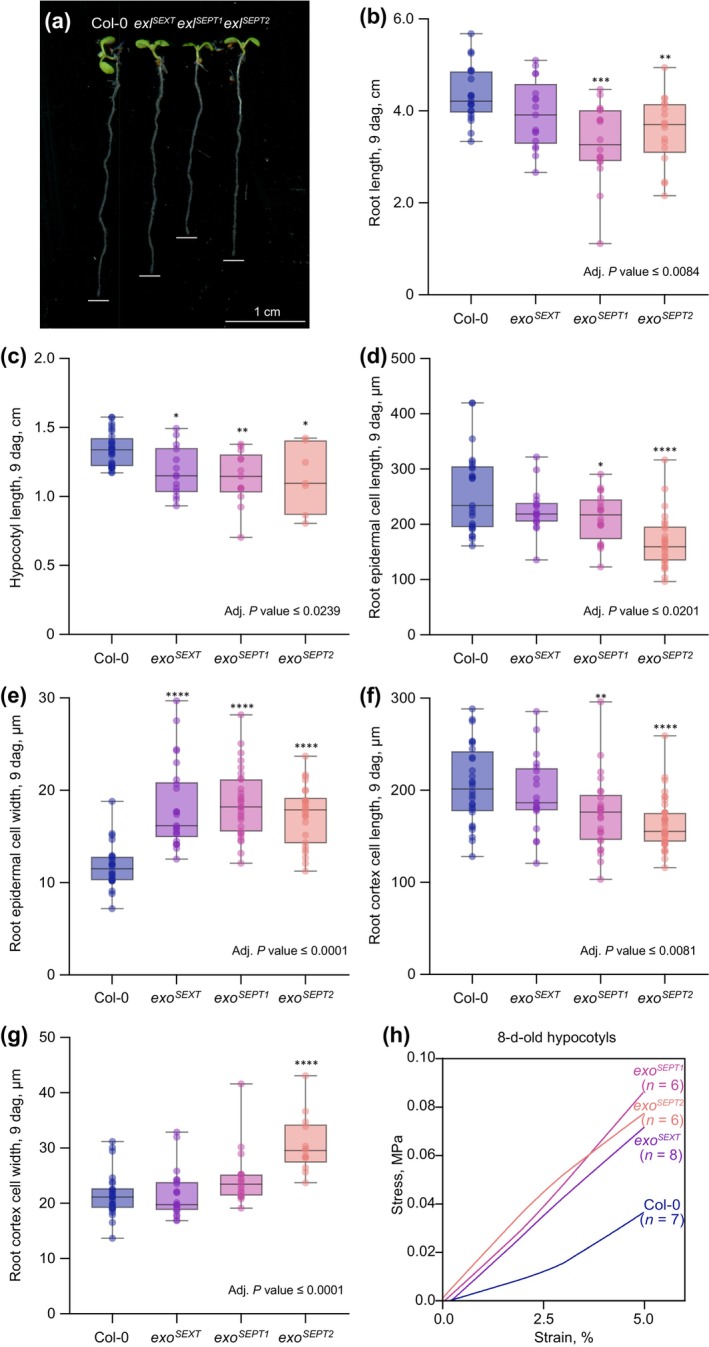
Phenotypes of *exo/exl* multiple mutants. (a) Representative images of seedlings of the indicated genotypes (8 dag). Root tip positions are marked by white bars. (b) Root length measurements. (c) Hypocotyl length measurements (7 dag, dark‐grown). (d–g) Quantification of epidermal (d, e) and cortex (f, g) cell length (d, f) and width (e, g). (h) Microextensometer force–displacement curves of dark‐grown hypocotyls, smoothed average, corrected for sample thickness. *exo* ^*SEXT*^: *exo‐2/exl1/2/3/4/6* sextuple mutant; *exo* ^*SEPT1*^ and *exo* ^*SEPT2*^: two independent *exo‐2/exl2/3/4/5/6/7* septuple mutants; statistically significant differences (asterisks) compared to Col‐0 were determined by ordinary one‐way ANOVA followed by Tukey's multiple comparison test, two‐sided. Seedlings were grown on ½ MS media supplemented with 0.3% sucrose, except for the hypocotyl assay in (c), which was performed on media without sucrose. Box plots display 2^nd^ and 3^rd^ quartiles and the median, whiskers indicate maximum and minimum. *, *P* ≦ 0.05; **, *P* ≦ 0.01; ***, *P* ≦ 0.001; ****, *P* ≦ 0.0001.

To unveil any *EXO/EXL*‐related phenotypes, we next built up multiple mutants of the gene family by CRISPR/Cas9‐mediated gene editing in the *exo‐2* mutant background. We thereby introduced mutations into all *EXO/EXL* genes, which can be considered loss‐of‐function alleles since they either carry nucleotide insertions or deletions that lead to frameshifts (*EXL1*, *EXL2*, *EXL3*, *EXL4*, *EXL6*), or large insertions or deletions (*EXL5*, *EXL7*) (Fig. S7). Again, we did not observe any apparent quantitatively robust mesoscopic phenotypes in lower order mutant combinations. However, visually obvious and statistically significant root and hypocotyl elongation phenotypes were observed in *exo‐2/exl1/2/3/4/6* sextuple (*exo* ^*SEXT*^) mutants as well as in two independent *exo‐2/exl2/3/4/5/6/7* septuple (*exo* ^*SEPT1*^ and *exo* ^*SEPT2*^) mutant lines (Fig. 7a). While root elongation was reduced up to *c*. 24% (Fig. 7b), hypocotyl elongation was reduced up to *c*. 14% (Fig. 7c). Despite repeated efforts, we could not obtain octuple mutants for complete knockout of the gene family. Matching the mesoscopic growth measurements, the *exo* ^*SEXT*^ and *exo* ^*SEPT*^ mutants also displayed shorter and wider cells in the root (Fig. 7d–g), suggesting that similar to the *agp* mutants, their reduced root and hypocotyl elongation reflects an underlying reduction in anisotropic cell expansion.

### *exo/exl* loss‐of‐function mutants display stiffer hypocotyls

To assess whether the *EXO/EXL* loss‐of‐function also had an impact on the mechanical properties of the cell wall, we again employed the microextensometer setup (Majda *et al*., 2022; Trozzi *et al*., 2025) to measure Young's modulus in the *exo* ^*SEXT*^ and *exo* ^*SEPT*^ mutants. We restricted these experiments to 8‐d‐old dark‐grown hypocotyls because this material proved to be technically most feasible and robust. Again, the measurements revealed that substantially more force had to be applied for the initial elastic extension of *exo* ^*SEXT*^ and *exo* ^*SEPT*^ mutants as compared to WT (Fig. 7h), indicating stiffer hypocotyls in the mutants. In summary, our phenotypic characterizations suggest that loss‐of‐function of selected *AGP* genes and *EXO/EXL* genes leads to comparable defects, which are however quantitatively somewhat less pronounced in *exo/exl* mutants.

### *agp* and *exo/exl* mutants show reduced brassinosteroid‐induced root elongation

Finally, we assayed whether *agp* and *exo/exl* mutants are impaired in their brassinosteroid growth response. Brassinosteroid application in tissue culture generally inhibits root growth of WT seedlings; however, low levels can slightly promote root growth (Mussig *et al*., 2003; Kang *et al*., 2017). We indeed observed significantly enhanced WT root elongation on low brassinolide concentration, whereas *agp* and *exo/exl* mutants did not respond (Fig. S9a). Moreover, when seedlings were sensitized to the treatment by first germinating and growing them on brassinazole, *agp* and *exo/exl* mutants appeared hypersensitive to brassinazole treatment, and the growth acceleration observed in WT after transfer on brassinolide was markedly reduced in the mutants (Fig. S9b,c). These results are consistent with the idea that *AGP* and *EXO/EXL* genes are required for a comprehensive brassinosteroid growth response.

## Discussion

Collectively, our results support our hypothesis at the outset that *AGP* and *EXO/EXL* genes are required for comprehensive anisotropic cell elongation. While their quantitative impact as judged from the mutants is comparatively mild, we cannot exclude that stronger phenotypes are masked by compensatory expression of other gene family members. For example, expression of the still intact *EXL1* gene appeared somewhat increased in the *exo* ^*SEPT2*^ mutant, although no expression changes were detected for the intact *EXL5* and *EXL7* genes in the *exo* ^*SEXT*^ mutant (Fig. S10). Moreover, we cannot strictly exclude off‐target effects in our CRISPR/Cas9‐generated mutants, but the fact that the phenotype was observed in independently produced mutant combinations and quantitatively aggravated as more genes were knocked out supports the hypothesis that it is genuine. Notably, we were not successful in constructing higher order mutants beyond the ones we report here for both *agp* and *exo/exl* genes, which may point to eventual viability or reproduction problems, as observed for the combinatorial *agp6 agp11* mutant but also the *agp4* mutant (Coimbra *et al*., 2009; Pereira *et al*., 2016).

Among the large gene families in plants, *AGPs* are one of the most complex (Seifert & Roberts, 2007; Ma & Zhao, 2010; Ma & Johnson, 2023), possibly because they encode evolutionary ancient cell wall components (Herve *et al*., 2016). The relative paucity of single mutant phenotypes suggests extensive redundancy among *AGP* genes. Nevertheless, a multitude of developmental and physiological roles have been proposed for AGPs, mostly by deduction from gain‐of‐function and pharmacological inhibition approaches, or from the phenotypes of mutants in AGP‐modifying enzymes (Ellis *et al*., 2010; Hromadova *et al*., 2021; Ma & Johnson, 2023). In this context, the beta‐Yariv reagent (and its negative control, the alpha‐Yariv reagent), which precipitates AGPs and thus interferes with their function (Yariv *et al*., 1967; Nothnagel, 1997; Prerovska *et al*., 2021), as well as mutants in beta‐glucosyltransferases that are impaired in post‐translational modification and maturation of AGPs (Basu *et al*., 2013, 2015; Geshi *et al*., 2013; Dilokpimol *et al*., 2014; Ogawa‐Ohnishi & Matsubayashi, 2015; Ajayi *et al*., 2021; Kaur *et al*., 2022, 2023; Nibbering *et al*., 2022) proved to be particularly useful. For example, the phenotypes of higher order mutants in hydroxyproline‐galactosyltransferases (Hyp‐GALTs) that can catalyze the addition of the first galactose sugar to hydroxyproline residues in AGP protein backbones suggest that AGPs are essential for normal vegetative and reproductive growth (Kaur *et al*., 2022, 2023). However, although a *hypgalt* octuple mutant is viable and displays a range of growth‐related and reproductive phenotypes (Kaur *et al*., 2023), a single mutant in another GALT that elongates AGP side chains is embryo‐lethal (Geshi *et al*., 2013). Moreover, a double mutant in two other *GALT* genes implicated in AGP side chain elongation affects growth likely due to cellulose‐related primary and secondary cell wall defects (Nibbering *et al*., 2022). These observations point to either highly nested activity of AGP‐modifying enzymes or to flexible substrate specificities, including non‐AGP‐related activities (Egelund *et al*., 2010; Narciso *et al*., 2021; Petit *et al*., 2021).

In our study, we concentrated on *AGP* genes that we selected on the premise that they play a role in brassinosteroid‐dependent growth. Our results confirm the suspected redundancy and collectively suggest an important role of classical and peptide AGPs in anisotropic cell expansion in both root and shoot tissues. Their pronounced brassinosteroid response is thus coherent with the reported reduced cellular anisotropy in brassinosteroid pathway mutants (Fridman *et al*., 2021; Graeff *et al*., 2021). Moreover, because the modification of pectin architecture triggers brassinosteroid signaling (Wolf *et al*., 2014) and because brassinosteroid signaling in turn modulates cell wall architecture (Rao & Dixon, 2017; Percio *et al*., 2024), the reduced pectin complexity in our *agp* mutants may mean that AGPs act as intermediate feedback regulators. Since the state of the pectin network has a pivotal influence on mechanical characteristics of the cell wall (Peaucelle *et al*., 2011; Senechal *et al*., 2024), it also appears possible that the increased organ stiffness in *agp* mutants largely reflects the observed changes in pectin complexity. The reported covalent AGP links with pectin (Tan *et al*., 2024) notwithstanding, the observed effects could also be indirect. AGPs can bind calcium in a pH‐dependent manner and thereby operate as reversible calcium capacitators (Lamport & Varnai, 2013; Lopez‐Hernandez *et al*., 2020). Reduced AGP levels may therefore indirectly alter cell wall mechanics by affecting calcium cross‐linking of pectins, which would also explain the difference in COS488 staining (Chebli & Geitmann, 2017).

Compared with the *agp* mutants, the *exo/exl* mutant phenotypes were quantitatively somewhat less pronounced and only observed once nearly the entire gene family was knocked out. Given the reported growth phenotype of the *exo‐1* single mutant (Schroder *et al*., 2009), this was surprising; however, our observations are in line with another report that did not observe any phenotypes upon *EXO* downregulation (Farrar *et al*., 2003). Differences in growth conditions (although we used largely similar parameters) could be responsible for this discrepancy, as well as other confounding factors such as background mutations or asynchronously produced seed stocks. Irrespectively, *EXO* is the most widely and among the stronger expressed family members, and collectively, *EXO/EXL* expression was observed across all root and hypocotyl tissues. The functional significance of EXO/EXL proteins remains largely enigmatic. Although there is evidence that they are secreted but likely not anchored to the plasma membrane (Farrar *et al*., 2003; Schroder *et al*., 2009, 2012), their biochemical activity or structural role is unknown. Our results suggest not only that EXO/EXL proteins could indeed play a role in brassinosteroid‐mediated anisotropic cell expansion but also that this role would be quantitatively minor as compared to AGPs in unchallenged conditions. It is conceivable then that the brassinosteroid pathway promotes anisotropic cell growth via multiple effector classes, which may on the one hand confer robustness to the network output but on the other hand also enhance fine‐tuning capacity.

Finally, in this initial report, we describe robust and visually obvious morphological and mechanical phenotypes in favorable growth conditions. Given the role of the brassinosteroid pathway in mediating stress responses (Planas‐Riverola *et al*., 2019; Nolan *et al*., 2020), it appears likely that other cellular‐level and/or physiological *AGP*‐ or *EXO/EXL*‐related phenotypes remain to be uncovered. The mutants produced for this study may serve to further explore this notion.

## Competing interests

None declared.

## Author contributions

DN and CSH designed the project and drafted the manuscript. DN, HND‐A, SR, KL, NT and LAB performed experiments and analyzed data. MM, TH and CSH supervised the work. All authors contributed to the assembly and the completion of the manuscript.

## Disclaimer

The New Phytologist Foundation remains neutral with regard to jurisdictional claims in maps and in any institutional affiliations.

## Supporting information


**Dataset S1** *AGP* gene expression data from single‐cell and bulk RNA sequencing experiments.

**Dataset S2** *EXO/EXL* gene expression data from single‐cell and bulk RNA sequencing experiments.

**Dataset S3** Oligonucleotides and sgRNAs used in this study.

**Fig. S1** Expression of many *AGP* genes is brassinosteroid‐dependent.
**Fig. S2** Expression patterns of *AGP* genes.
**Fig. S3** CRISPR/Cas9‐generated mutants in *AGP* genes.
**Fig. S4** *agp* multiple mutant phenotypes.
**Fig. S5** Mechanical properties of *agp* multiple mutants.
**Fig. S6** Phenotypes of *exo‐1* mutants.
**Fig. S7** CRISPR/Cas9‐generated mutants in *EXO/EXL* genes.
**Fig. S8** Expression patterns of *EXO/EXL* genes.
**Fig. S9** Brassinosteroid response of *agp* and *exo/exl* mutants.
**Fig. S10** Expression levels of *EXL* genes.
Please note: Wiley is not responsible for the content or functionality of any Supporting Information supplied by the authors. Any queries (other than missing material) should be directed to the *New Phytologist* Central Office.

## Data Availability

The data that support the findings of this study are available in the Datasets S1–, S3. Accessions nos.: The IDs for the targeted genes are *AGP2* (AT2G22470), *AGP4* (AT5G10430), *AGP10* (AT4G09030), *AGP11* (AT3G01700), *AGP13* (AT4G26320), *AGP22* (AT5G53250), *AGP24* (AT5G40730), *EXO* (AT4G08950), *EXL1* (AT1G35140), *EXL2* (AT5G64260), *EXL3* (AT5G51550), *EXL4* (AT5G09440), *EXL5* (AT2G17230), *EXL6* (AT3G02970), *EXL7* (AT2G35150).

## References

[nph71063-bib-0001] Ajayi OO , Held MA , Showalter AM . 2021. Three beta‐glucuronosyltransferase genes involved in arabinogalactan biosynthesis function in Arabidopsis growth and development. Plants 10: 1172. 34207602 10.3390/plants10061172 PMC8227792

[nph71063-bib-0002] Alonso Baez L , Bacete L . 2023. Cell wall dynamics: novel tools and research questions. Journal of Experimental Botany 74: 6448–6467. 37539735 10.1093/jxb/erad310 PMC10662238

[nph71063-bib-0003] Bacete L , Schulz J , Engelsdorf T , Bartosova Z , Vaahtera L , Yan G , Gerhold JM , Ticha T , Ovstebo C , Gigli‐Bisceglia N *et al*. 2022. THESEUS1 modulates cell wall stiffness and abscisic acid production in *Arabidopsis thaliana* . Proceedings of the National Academy of Sciences, USA 119: e2119258119. 10.1073/pnas.2119258119 PMC8740707 34949719

[nph71063-bib-0004] Bartels D , Classen B . 2017. Structural investigations on arabinogalactan‐proteins from a lycophyte and different monilophytes (ferns) in the evolutionary context. Carbohydrate Polymers 172: 342–351. 28606543 10.1016/j.carbpol.2017.05.031

[nph71063-bib-0005] Basu D , Liang Y , Liu X , Himmeldirk K , Faik A , Kieliszewski M , Held M , Showalter AM . 2013. Functional identification of a hydroxyproline‐o‐galactosyltransferase specific for arabinogalactan protein biosynthesis in Arabidopsis. The Journal of Biological Chemistry 288: 10132–10143. 23430255 10.1074/jbc.M112.432609 PMC3617256

[nph71063-bib-0006] Basu D , Tian L , Wang W , Bobbs S , Herock H , Travers A , Showalter AM . 2015. A small multigene hydroxyproline‐O‐galactosyltransferase family functions in arabinogalactan‐protein glycosylation, growth and development in Arabidopsis. BMC Plant Biology 15: 295. 26690932 10.1186/s12870-015-0670-7 PMC4687291

[nph71063-bib-0007] Borassi C , Gloazzo Dorosz J , Ricardi MM , Carignani Sardoy M , Pol Fachin L , Marzol E , Mangano S , Rodriguez Garcia DR , Martinez Pacheco J , Rondon Guerrero YDC *et al*. 2020. A cell surface arabinogalactan‐peptide influences root hair cell fate. New Phytologist 227: 732–743. 32064614 10.1111/nph.16487

[nph71063-bib-0008] Chaiwanon J , Wang ZY . 2015. Spatiotemporal brassinosteroid signaling and antagonism with auxin pattern stem cell dynamics in Arabidopsis roots. Current Biology 25: 1031–1042. 25866388 10.1016/j.cub.2015.02.046 PMC4415608

[nph71063-bib-0009] Chebli Y , Geitmann A . 2017. Cellular growth in plants requires regulation of cell wall biochemistry. Current Opinion in Cell Biology 44: 28–35. 28131101 10.1016/j.ceb.2017.01.002

[nph71063-bib-0010] Clark NM , Nolan TM , Wang P , Song G , Montes C , Valentine CT , Guo H , Sozzani R , Yin Y , Walley JW . 2021. Integrated omics networks reveal the temporal signaling events of brassinosteroid response in Arabidopsis. Nature Communications 12: 5858. 10.1038/s41467-021-26165-3 PMC8494934 34615886

[nph71063-bib-0011] Coimbra S , Costa M , Jones B , Mendes MA , Pereira LG . 2009. Pollen grain development is compromised in Arabidopsis agp6 agp11 null mutants. Journal of Experimental Botany 60: 3133–3142. 19433479 10.1093/jxb/erp148 PMC2718217

[nph71063-bib-0012] Dilokpimol A , Poulsen CP , Vereb G , Kaneko S , Schulz A , Geshi N . 2014. Galactosyltransferases from *Arabidopsis thaliana* in the biosynthesis of type II arabinogalactan: molecular interaction enhances enzyme activity. BMC Plant Biology 14: 90. 24693939 10.1186/1471-2229-14-90 PMC4234293

[nph71063-bib-0013] Edri E , Cooper JK , Sharp ID , Guldi DM , Frei H . 2017. Ultrafast charge transfer between light absorber and Co(3)O(4) water oxidation catalyst across molecular wires embedded in silica membrane. Journal of the American Chemical Society 139: 5458–5466. 28355079 10.1021/jacs.7b01070

[nph71063-bib-0014] Egelund J , Ellis M , Doblin M , Qu Y , Bacic A . 2010. Genes and enzymes of the GT31 family: towards unravelling the function(s) of the plant glycosyltransferase family members. Annual Plant Reviews: Plant Polysaccharides, Biosynthesis and Bioengineering 41: 213–234.

[nph71063-bib-0015] Ellis M , Egelund J , Schultz CJ , Bacic A . 2010. Arabinogalactan‐proteins: key regulators at the cell surface? Plant Physiology 153: 403–419. 20388666 10.1104/pp.110.156000 PMC2879789

[nph71063-bib-0016] Farrar K , Evans IM , Topping JF , Souter MA , Nielsen JE , Lindsey K . 2003. EXORDIUM‐‐a gene expressed in proliferating cells and with a role in meristem function, identified by promoter trapping in Arabidopsis. The Plant Journal 33: 61–73. 12943541 10.1046/j.1365-313x.2003.01608.x

[nph71063-bib-0017] Fridman Y , Strauss S , Horev G , Ackerman‐Lavert M , Reiner‐Benaim A , Lane B , Smith RS , Savaldi‐Goldstein S . 2021. The root meristem is shaped by brassinosteroid control of cell geometry. Nature Plants 7: 1475–1484. 34782771 10.1038/s41477-021-01014-9 PMC8592843

[nph71063-bib-0018] Geshi N , Johansen JN , Dilokpimol A , Rolland A , Belcram K , Verger S , Kotake T , Tsumuraya Y , Kaneko S , Tryfona T *et al*. 2013. A galactosyltransferase acting on arabinogalactan protein glycans is essential for embryo development in Arabidopsis. The Plant Journal 76: 128–137. 23837821 10.1111/tpj.12281

[nph71063-bib-0019] Graeff M , Rana S , Wendrich JR , Dorier J , Eekhout T , Aliaga Fandino AC , Guex N , Bassel GW , De Rybel B , Hardtke CS . 2021. A single‐cell morpho‐transcriptomic map of brassinosteroid action in the Arabidopsis root. Molecular Plant 14: 1985–1999. 34358681 10.1016/j.molp.2021.07.021 PMC8674818

[nph71063-bib-0020] Herve C , Simeon A , Jam M , Cassin A , Johnson KL , Salmean AA , Willats WG , Doblin MS , Bacic A , Kloareg B . 2016. Arabinogalactan proteins have deep roots in eukaryotes: identification of genes and epitopes in brown algae and their role in *Fucus serratus* embryo development. New Phytologist 209: 1428–1441. 26667994 10.1111/nph.13786

[nph71063-bib-0021] Hromadova D , Soukup A , Tylova E . 2021. Arabinogalactan proteins in plant roots – an update on possible functions. Frontiers in Plant Science 12: 674010. 34079573 10.3389/fpls.2021.674010 PMC8165308

[nph71063-bib-0022] Jonsson K , Hamant O , Bhalerao RP . 2022. Plant cell walls as mechanical signaling hubs for morphogenesis. Current Biology 32: R334–R340. 35413265 10.1016/j.cub.2022.02.036

[nph71063-bib-0023] Kang YH , Breda A , Hardtke CS . 2017. Brassinosteroid signaling directs formative cell divisions and protophloem differentiation in Arabidopsis root meristems. Development 144: 272–280. 28096215 10.1242/dev.145623 PMC5394764

[nph71063-bib-0024] Kaur D , Held MA , Zhang Y , Moreira D , Coimbra S , Showalter AM . 2023. Knockout of eight hydroxyproline‐O‐galactosyltransferases cause multiple vegetative and reproductive growth defects. Cell Surface 10: 100117. 38076635 10.1016/j.tcsw.2023.100117 PMC10698532

[nph71063-bib-0025] Kaur D , Moreira D , Coimbra S , Showalter AM . 2022. Hydroxyproline‐O‐galactosyltransferases synthesizing type II arabinogalactans are essential for male gametophytic development in Arabidopsis. Frontiers in Plant Science 13: 935413. 35774810 10.3389/fpls.2022.935413 PMC9237623

[nph71063-bib-0026] Kim EJ , Russinova E . 2020. Brassinosteroid signalling. Current Biology 30: R294–R298. 32259497 10.1016/j.cub.2020.02.011

[nph71063-bib-0027] Kim TW , Guan S , Sun Y , Deng Z , Tang W , Shang JX , Sun Y , Burlingame AL , Wang ZY . 2009. Brassinosteroid signal transduction from cell‐surface receptor kinases to nuclear transcription factors. Nature Cell Biology 11: 1254–1260. 19734888 10.1038/ncb1970 PMC2910619

[nph71063-bib-0028] Kohnen MV , Schmid‐Siegert E , Trevisan M , Petrolati LA , Senechal F , Muller‐Moule P , Maloof J , Xenarios I , Fankhauser C . 2016. Neighbor detection induces organ‐specific transcriptomes, revealing patterns underlying hypocotyl‐specific growth. Plant Cell 28: 2889–2904. 27923878 10.1105/tpc.16.00463 PMC5240736

[nph71063-bib-0029] Kurihara D , Mizuta Y , Sato Y , Higashiyama T . 2015. ClearSee: a rapid optical clearing reagent for whole‐plant fluorescence imaging. Development 142: 4168–4179. 26493404 10.1242/dev.127613 PMC4712841

[nph71063-bib-0030] Lamport DTA , Varnai P . 2013. Periplasmic arabinogalactan glycoproteins act as a calcium capacitor that regulates plant growth and development. New Phytologist 197: 58–64. 23106282 10.1111/nph.12005

[nph71063-bib-0031] Lee KJ , Sakata Y , Mau SL , Pettolino F , Bacic A , Quatrano RS , Knight CD , Knox JP . 2005. Arabinogalactan proteins are required for apical cell extension in the moss Physcomitrella patens. Plant Cell 17: 3051–3065. 16199618 10.1105/tpc.105.034413 PMC1276029

[nph71063-bib-0032] Lopez‐Hernandez F , Tryfona T , Rizza A , Yu XL , Harris MOB , Webb AAR , Kotake T , Dupree P . 2020. Calcium binding by arabinogalactan polysaccharides is important for normal plant development. Plant Cell 32: 3346–3369. 32769130 10.1105/tpc.20.00027 PMC7534474

[nph71063-bib-0033] Ma H , Zhao J . 2010. Genome‐wide identification, classification, and expression analysis of the arabinogalactan protein gene family in rice (*Oryza sativa* L.). Journal of Experimental Botany 61: 2647–2668. 20423940 10.1093/jxb/erq104 PMC2882264

[nph71063-bib-0034] Ma Y , Johnson K . 2023. Arabinogalactan proteins – multifunctional glycoproteins of the plant cell wall. Cell Surface 9: 100102. 36873729 10.1016/j.tcsw.2023.100102 PMC9974416

[nph71063-bib-0035] Ma Y , Yan C , Li H , Wu W , Liu Y , Wang Y , Chen Q , Ma H . 2017. Bioinformatics prediction and evolution analysis of arabinogalactan proteins in the plant kingdom. Frontiers in Plant Science 8: 66. 28184232 10.3389/fpls.2017.00066 PMC5266747

[nph71063-bib-0036] Majda M , Trozzi N , Mosca G , Smith RS . 2022. How cell geometry and cellular patterning influence tissue stiffness. International Journal of Molecular Sciences 23: 5651. 35628463 10.3390/ijms23105651 PMC9145195

[nph71063-bib-0037] Mravec J , Kracun SK , Rydahl MG , Westereng B , Miart F , Clausen MH , Fangel JU , Daugaard M , Van Cutsem P , De Fine Licht HH *et al*. 2014. Tracking developmentally regulated post‐synthetic processing of homogalacturonan and chitin using reciprocal oligosaccharide probes. Development 141: 4841–4850. 25395456 10.1242/dev.113365

[nph71063-bib-0038] Mravec J , Kracun SK , Rydahl MG , Westereng B , Pontiggia D , De Lorenzo G , Domozych DS , Willats WGT . 2017. An oligogalacturonide‐derived molecular probe demonstrates the dynamics of calcium‐mediated pectin complexation in cell walls of tip‐growing structures. The Plant Journal 91: 534–546. 28419587 10.1111/tpj.13574

[nph71063-bib-0039] Mueller KK , Pfeifer L , Schuldt L , Szovenyi P , de Vries S , de Vries J , Johnson KL , Classen B . 2023. Fern cell walls and the evolution of arabinogalactan proteins in streptophytes. The Plant Journal 114: 875–894. 36891885 10.1111/tpj.16178

[nph71063-bib-0040] Muniz M , Riezman H . 2016. Trafficking of glycosylphosphatidylinositol anchored proteins from the endoplasmic reticulum to the cell surface. Journal of Lipid Research 57: 352–360. 26450970 10.1194/jlr.R062760 PMC4767001

[nph71063-bib-0041] Mussig C , Shin GH , Altmann T . 2003. Brassinosteroids promote root growth in Arabidopsis. Plant Physiology 133: 1261–1271. 14526105 10.1104/pp.103.028662 PMC281621

[nph71063-bib-0042] Narciso JO , Zeng W , Ford K , Lampugnani ER , Humphries J , Austarheim I , van de Meene A , Bacic A , Doblin MS . 2021. Biochemical and functional characterization of GALT8, an Arabidopsis GT31 beta‐(1,3)‐galactosyltransferase that influences seedling development. Frontiers in Plant Science 12: 678564. 34113372 10.3389/fpls.2021.678564 PMC8186459

[nph71063-bib-0043] Nibbering P , Castilleux R , Wingsle G , Niittyla T . 2022. CAGEs are golgi‐localized GT31 enzymes involved in cellulose biosynthesis in Arabidopsis. The Plant Journal 110: 1271–1285. 35289007 10.1111/tpj.15734 PMC9321575

[nph71063-bib-0044] Nolan TM , Vukasinovic N , Hsu CW , Zhang J , Vanhoutte I , Shahan R , Taylor IW , Greenstreet L , Heitz M , Afanassiev A *et al*. 2023. Brassinosteroid gene regulatory networks at cellular resolution in the Arabidopsis root. Science 379: eadf4721. 36996230 10.1126/science.adf4721 PMC10119888

[nph71063-bib-0045] Nolan TM , Vukasinovic N , Liu D , Russinova E , Yin Y . 2020. Brassinosteroids: multidimensional regulators of plant growth, development, and stress responses. Plant Cell 32: 295–318. 31776234 10.1105/tpc.19.00335 PMC7008487

[nph71063-bib-0046] Nothnagel EA . 1997. Proteoglycans and related components in plant cells. International Review of Cytology 174: 195–291. 9161008 10.1016/s0074-7696(08)62118-x

[nph71063-bib-0047] Ogawa‐Ohnishi M , Matsubayashi Y . 2015. Identification of three potent hydroxyproline O‐galactosyltransferases in Arabidopsis. The Plant Journal 81: 736–746. 25600942 10.1111/tpj.12764

[nph71063-bib-0048] Pacheco‐Villalobos D , Diaz‐Moreno SM , van der Schuren A , Tamaki T , Kang YH , Gujas B , Novak O , Jaspert N , Li Z , Wolf S *et al*. 2016. The effects of high steady state auxin levels on root cell elongation in *Brachypodium* . Plant Cell 28: 1009–1024. 27169463 10.1105/tpc.15.01057 PMC4904674

[nph71063-bib-0049] Park YB , Cosgrove DJ . 2012. Changes in cell wall biomechanical properties in the xyloglucan‐deficient xxt1/xxt2 mutant of Arabidopsis. Plant Physiology 158: 465–475. 22108526 10.1104/pp.111.189779 PMC3252101

[nph71063-bib-0050] Peaucelle A , Braybrook SA , Le Guillou L , Bron E , Kuhlemeier C , Hofte H . 2011. Pectin‐induced changes in cell wall mechanics underlie organ initiation in Arabidopsis. Current Biology 21: 1720–1726. 21982593 10.1016/j.cub.2011.08.057

[nph71063-bib-0051] Percio F , Rubio L , Amorim‐Silva V , Botella MA . 2024. Crucial roles of brassinosteroids in cell wall composition and structure across species: new insights and biotechnological applications. Plant, Cell & Environment 48: 1751–1767. 10.1111/pce.15258 PMC11788965 39491539

[nph71063-bib-0052] Pereira AM , Nobre MS , Pinto SC , Lopes AL , Costa ML , Masiero S , Coimbra S . 2016. Love is strong, and you're so sweet: JAGGER is essential for persistent synergid degeneration and polytubey block in *Arabidopsis thaliana* . Molecular Plant 9: 601–614. 26774620 10.1016/j.molp.2016.01.002

[nph71063-bib-0053] Petit D , Teppa RE , Harduin‐Lepers A . 2021. A phylogenetic view and functional annotation of the animal beta1,3‐glycosyltransferases of the GT31 CAZy family. Glycobiology 31: 243–259. 32886776 10.1093/glycob/cwaa086 PMC8022947

[nph71063-bib-0054] Planas‐Riverola A , Gupta A , Betegon‐Putze I , Bosch N , Ibanes M , Cano‐Delgado AI . 2019. Brassinosteroid signaling in plant development and adaptation to stress. Development 146: dev151894. 30872266 10.1242/dev.151894 PMC6432667

[nph71063-bib-0055] Prerovska T , Pavlu A , Hancharyk D , Rodionova A , Vavrikova A , Spiwok V . 2021. Structural basis of the function of Yariv reagent‐an important tool to study arabinogalactan proteins. Frontiers in Molecular Biosciences 8: 682858. 34179088 10.3389/fmolb.2021.682858 PMC8230119

[nph71063-bib-0056] Rao X , Dixon RA . 2017. Brassinosteroid mediated cell wall remodeling in grasses under abiotic stress. Frontiers in Plant Science 8: 806. 28567047 10.3389/fpls.2017.00806 PMC5434148

[nph71063-bib-0057] Schroder F , Lisso J , Lange P , Mussig C . 2009. The extracellular EXO protein mediates cell expansion in Arabidopsis leaves. BMC Plant Biology 9: 20. 19216774 10.1186/1471-2229-9-20 PMC2661892

[nph71063-bib-0058] Schroder F , Lisso J , Mussig C . 2012. Expression pattern and putative function of EXL1 and homologous genes in Arabidopsis. Plant Signaling & Behavior 7: 22–27. 22301961 10.4161/psb.7.1.18369 PMC3357360

[nph71063-bib-0059] Schultz CJ , Johnson KL , Currie G , Bacic A . 2000. The classical arabinogalactan protein gene family of Arabidopsis. Plant Cell 12: 1751–1768. 11006345 10.1105/tpc.12.9.1751 PMC149083

[nph71063-bib-0060] Seifert GJ , Roberts K . 2007. The biology of arabinogalactan proteins. Annual Review of Plant Biology 58: 137–161. 10.1146/annurev.arplant.58.032806.103801 17201686

[nph71063-bib-0061] Senechal F , Robinson S , Van Schaik E , Trevisan M , Saxena P , Reinhardt D , Fankhauser C . 2024. Pectin methylesterification state and cell wall mechanical properties contribute to neighbor proximity‐induced hypocotyl growth in Arabidopsis. Plant Direct 8: e584. 38646567 10.1002/pld3.584 PMC11033045

[nph71063-bib-0062] Shaw SL . 2019. Seeing the cell wall in a new light. Plant Physiology 181: 9–11. 31467140 10.1104/pp.19.00776 PMC6716248

[nph71063-bib-0063] Sherrier DJ , Prime TA , Dupree P . 1999. Glycosylphosphatidylinositol‐anchored cell‐surface proteins from Arabidopsis. Electrophoresis 20: 2027–2035. 10451111 10.1002/(SICI)1522-2683(19990701)20:10<2027::AID-ELPS2027>3.0.CO;2-A

[nph71063-bib-0064] Silva J , Ferraz R , Dupree P , Showalter AM , Coimbra S . 2020. Three decades of advances in arabinogalactan‐protein biosynthesis. Frontiers in Plant Science 11: 610377. 33384708 10.3389/fpls.2020.610377 PMC7769824

[nph71063-bib-0065] Sun Y , Fan XY , Cao DM , Tang W , He K , Zhu JY , He JX , Bai MY , Zhu S , Oh E *et al*. 2010. Integration of brassinosteroid signal transduction with the transcription network for plant growth regulation in Arabidopsis. Developmental Cell 19: 765–777. 21074725 10.1016/j.devcel.2010.10.010 PMC3018842

[nph71063-bib-0066] Takahashi D , Soga K , Kikuchi T , Kutsuno T , Hao P , Sasaki K , Nishiyama Y , Kidokoro S , Sampathkumar A , Bacic A *et al*. 2024. Structural changes in cell wall pectic polymers contribute to freezing tolerance induced by cold acclimation in plants. Current Biology 34: 958–968. 38335960 10.1016/j.cub.2024.01.045

[nph71063-bib-0067] Tan L , Cheng J , Zhang L , Backe J , Urbanowicz B , Heiss C , Azadi P . 2024. Pectic‐AGP is a major form of Arabidopsis AGPs. Carbohydrate Polymers 330: 121838. 38368088 10.1016/j.carbpol.2024.121838

[nph71063-bib-0068] Trozzi N , Wodniok W , Kelly‐Bellow R , Meraviglia A , Chetelat A , Adkins N , Lane B , Smith RS , Kwiatkowska D , Majda M . 2025. Camelot: a computer‐automated micro‐extensometer with low‐cost optical tracking. BMC Biology 23: 112. 40289087 10.1186/s12915-025-02216-9 PMC12036183

[nph71063-bib-0069] Verhertbruggen Y , Marcus SE , Chen J , Knox JP . 2013. Cell wall pectic arabinans influence the mechanical properties of Arabidopsis thaliana inflorescence stems and their response to mechanical stress. Plant & Cell Physiology 54: 1278–1288. 23695504 10.1093/pcp/pct074

[nph71063-bib-0070] Wolf S , van der Does D , Ladwig F , Sticht C , Kolbeck A , Schurholz AK , Augustin S , Keinath N , Rausch T , Greiner S *et al*. 2014. A receptor‐like protein mediates the response to pectin modification by activating brassinosteroid signaling. Proceedings of the National Academy of Sciences, USA 111: 15261–15266. 10.1073/pnas.1322979111 PMC4210321 25288746

[nph71063-bib-0071] Wymer CL , Bibikova TN , Gilroy S . 1997. Cytoplasmic free calcium distributions during the development of root hairs of *Arabidopsis thaliana* . The Plant Journal 12: 427–439. 9301093 10.1046/j.1365-313x.1997.12020427.x

[nph71063-bib-0072] Yariv J , Lis H , Katchalski E . 1967. Precipitation of arabic acid and some seed polysaccharides by glycosylphenylazo dyes. The Biochemical Journal 105: 1C–2C. 6069833 10.1042/bj1050001c PMC1198314

[nph71063-bib-0073] Yu X , Li L , Zola J , Aluru M , Ye H , Foudree A , Guo H , Anderson S , Aluru S , Liu P *et al*. 2011. A brassinosteroid transcriptional network revealed by genome‐wide identification of BESI target genes in *Arabidopsis thaliana* . The Plant Journal 65: 634–646. 21214652 10.1111/j.1365-313X.2010.04449.x

[nph71063-bib-0074] Zhang J , Scarcelli G . 2021. Mapping mechanical properties of biological materials via an add‐on Brillouin module to confocal microscopes. Nature Protocols 16: 1251–1275. 33452504 10.1038/s41596-020-00457-2 PMC8218248

