# Supplementary material for: AGP and EXO‐LIKE genes promote brassinosteroid‐dependent anisotropic growth
Source: New Phytol. 2026 Mar 8;250(4):2384–98. doi: 10.1111/nph.71063 (PMC13103426; doi:10.1111/nph.71063)
Supplement: Supplementary file 4 — Fig. S1 Expression of many AGP genes is brassinosteroid‐dependent. Fig. S2 Expression patterns of AGP genes. Fig. S3 CRISPR/Cas9‐generated mutants in AGP genes. Fig. S4 agp multiple mutant phenotypes. Fig. S5 Mechanical properties of agp multiple mutants. Fig. S6 Phenotypes of exo‐1 mutants. Fig. S7 CRISPR/Cas9‐generated mutants in EXO/EXL genes. Fig. S8 Expression patterns of EXO/EXL genes. Fig. S9 Brassinosteroid response of agp and exo/exl mutants. Fig. S10 Expression levels of EXL genes. Please note: Wiley is not responsible for the content or functionality of any Supporting Information supplied by the authors. Any queries (other than missing material) should be directed to the New Phytologist Central Office. [file NPH-250-2384-s003.pdf]

New Phytologist Supporting Information

Article title: *AGP* and *EXO-LIKE* genes promote brassinosteroid-dependent anisotropic growth

Authors: Daria Novikova, Surbhi Rana, Kunkun Li, H. Nicholay Diaz-Ardila, Nicola Trozzi, Luis Alonso Baez, Thorsten Hamann, Mateusz Majda & Christian S. Hardtke

Article acceptance date: 15 February 2026

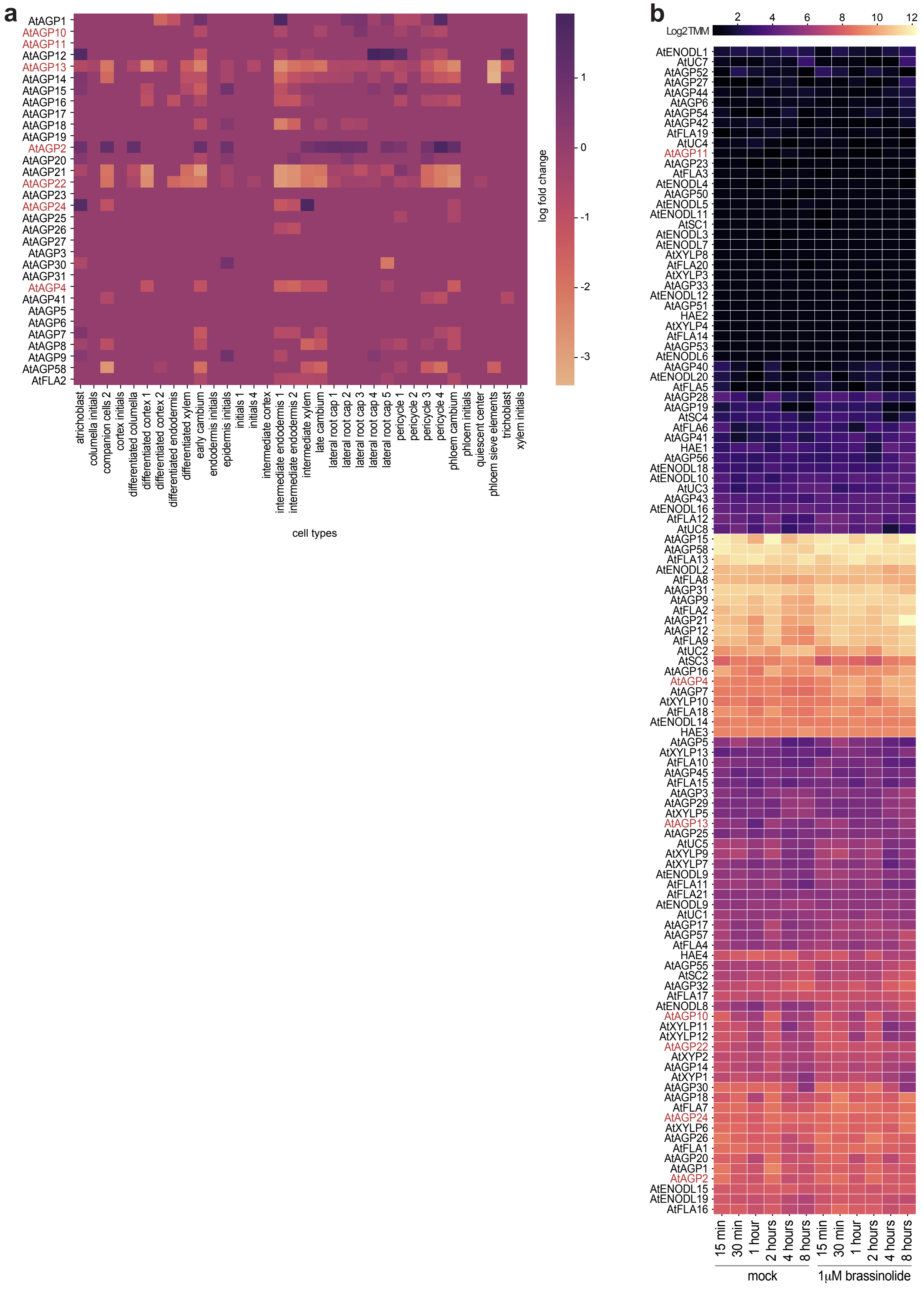

Supporting Figure S1: Expression of many *AGP* genes is brassinosteroid-dependent. (a) Heatmap of tissue-specific *AGP* gene expression (scRNAseq) in brassinosteroid triple receptor mutants as compared to wildtype (data from Graeff et al. 2021). **(b)** Heatmap of Arabidopsis *AGP* gene expression (RNAseq) upon brassinolide treatment of roots depleted of brassinosteroids by prior brassinazole (BRZ) treatment (data from Clark et al. 2021).

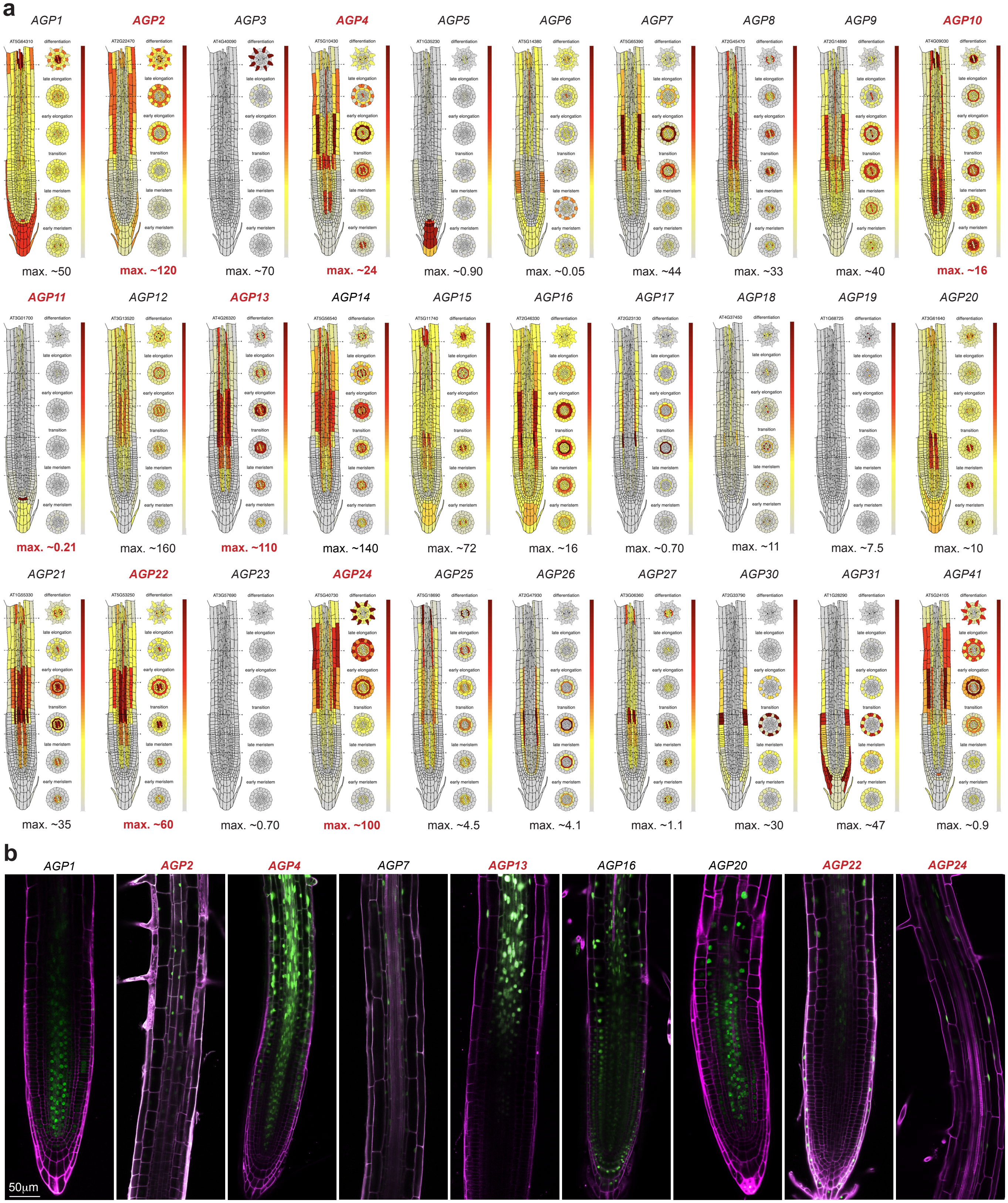

Supporting Figure S2: Expression patterns of *AGP* genes. (a) Schematic representation of root tip expression patterns of *AGP* genes, obtained from aggregation of multiple independent scRNAseq experiments of Arabidopsis Col-0 wildtype roots (https://rootcellatlas.org). Note the differences in expression level scales. **(b)** Confocal microscopy of root tips expressing transgenic transcriptional reporters (nuclear localized VENUS protein, green fluorescence) with promoters of the indicated *AGP* genes in wildtype (Col-0) background. Propidium iodide cell wall staining: magenta fluorescence. Genes for which mutants were obtained are highlighted in red.

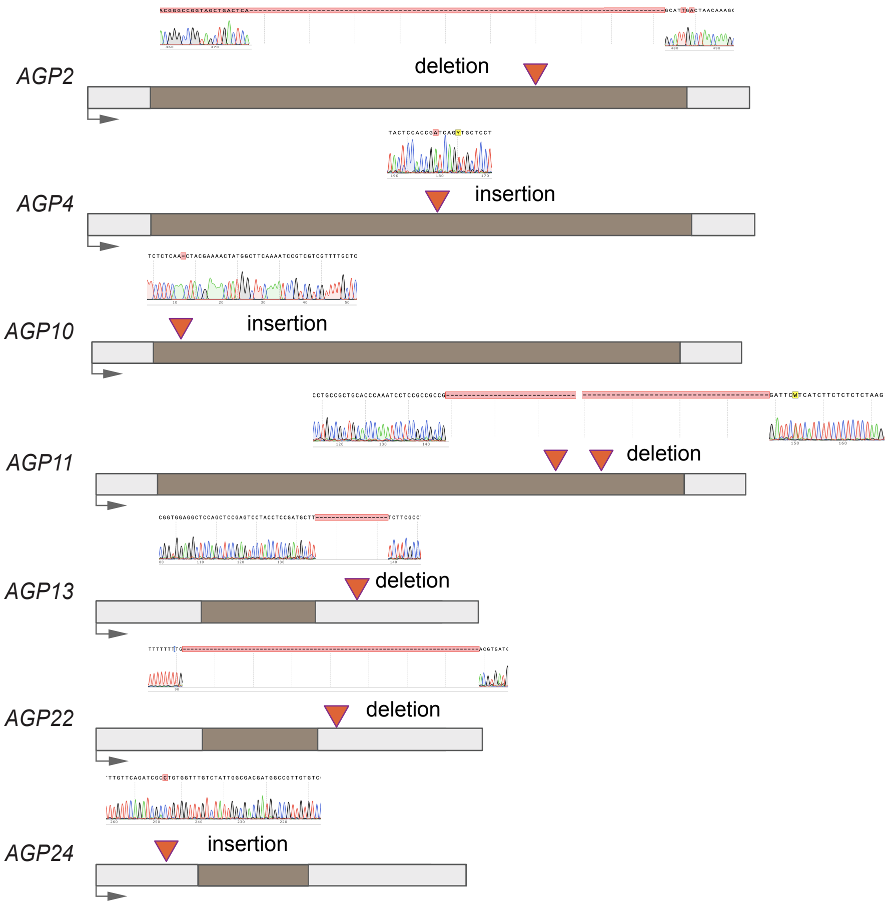

Supporting Figure S3: CRISPR/Cas9-generated mutants in *AGP* genes. Schematic representation of the prepropeptide open reading frames of *AGP* genes for which loss-of-function mutants were obtained. The mature AGP moieties are shaded in brown. Sequence traces of the CRISPR/Cas9-generated homozygous alleles are shown above each gene schematic.

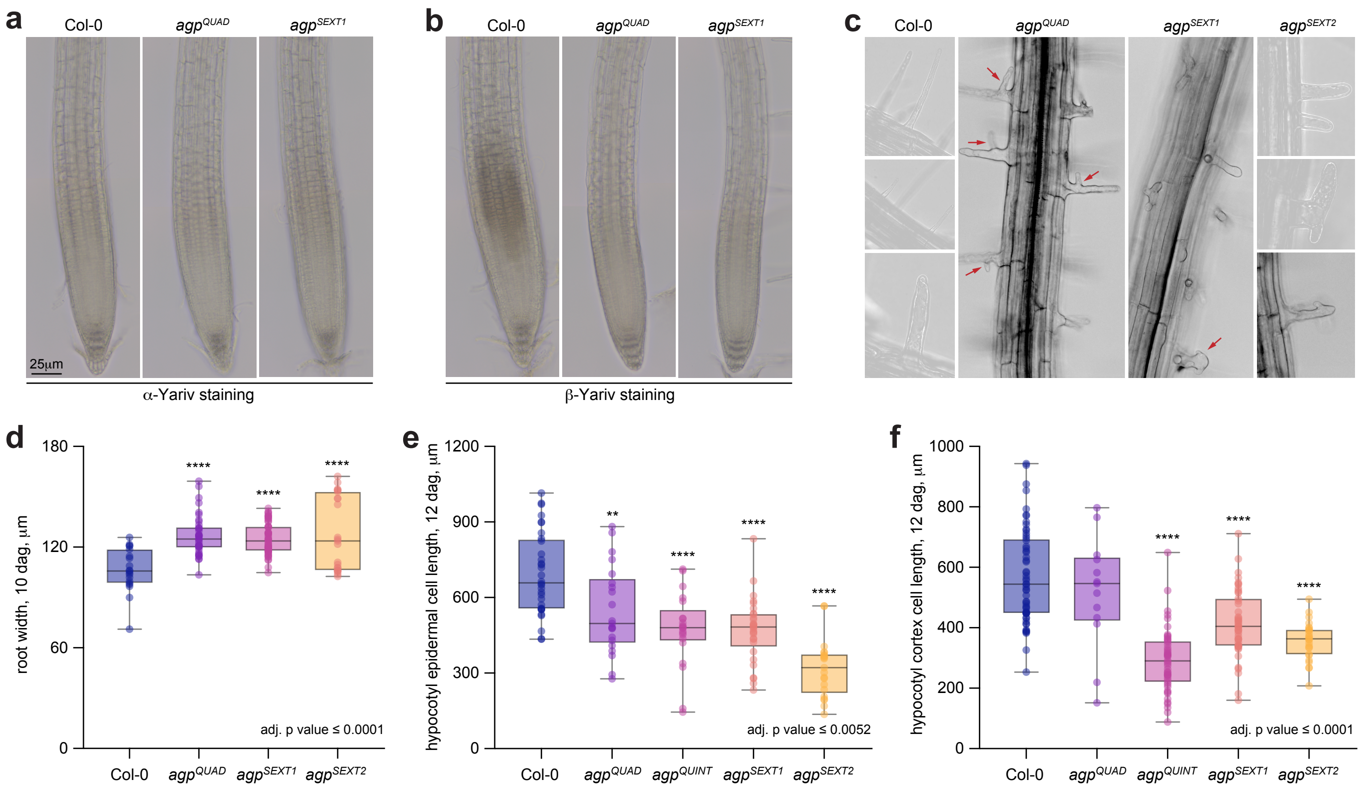

Supporting Figure S4: *agp* multiple mutant phenotypes. (a-b) Representative light microscopy images of roots stained with the AGP detecting beta-Yariv agent (b) or its alpha-Yariv control (a). **(c)** Light microscopy images (differential interference contrast) illustrating the range of branched or bulged root hair phenotypes in *agp* mutants. **(d)** Quantification of root width. **(e-f)** Quantification epidermal (e) and cortex (f) cell length in dark-grown hypocotyls (7 dag). Statistically significant differences (asterisks) compared to Col-0 were determined by ordinary one-way ANOVA followed by Tukey's multiple comparison test, two-sided. Box plots display 2nd and 3rd quartiles and the median, whiskers indicate maximum and minimum. **: $p \leqq 0.01$; ****: $p \leqq 0.0001$;

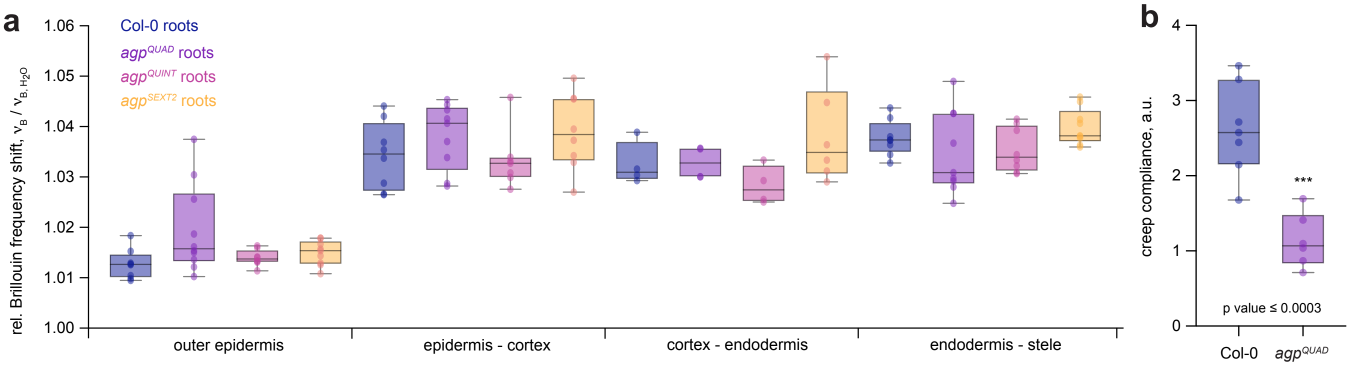

Supporting Figure S5: Mechanical properties of *agp* multiple mutants. (a) Brillouin microscopy measurements of elastic properties of individual cell walls in the ground tissues. **(b)** Creep compliance measurements for dark-grown Col-0 and mutant hypocotyls (7 dag). Statistically significant difference (asterisks) compared to Col-0 was determined by Student's t-test, two-sided. Box plots display 2nd and 3rd quartiles and the median, whiskers indicate maximum and minimum. ***: p≦0.001;

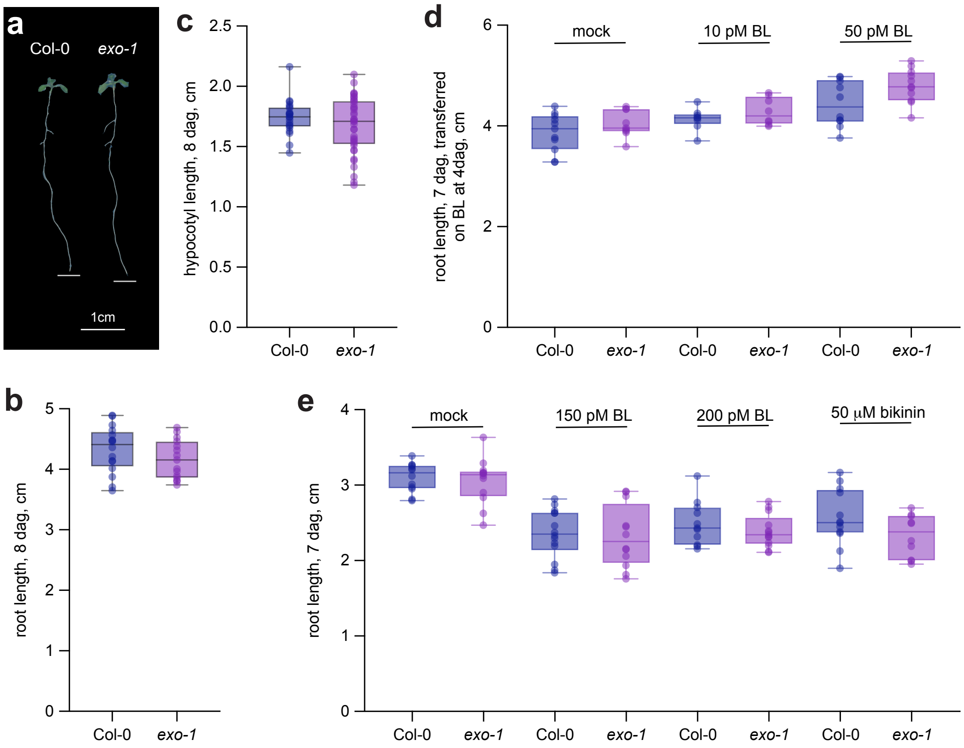

Supporting Figure S6: Phenotypes of *exo-1* mutants. (a) Representative images of seedlings of the indicated genotypes (6 dag). The *exo-1* line is line SALK_098602 and carries a homozygous T-DNA insertion in the coding sequence. Root tip positions are marked by white bars. **(b)** Root length measurements. **(c)** Hypocotyl length measurements (8 dag, dark-grown). **(d-e)** Quantification of root length in response to application of low brassinolide (BL) levels which slightly promote wildtype root growth (d), and in response to higher BL levels that inhibit root elongation and bikinin (a brassinosteroid signaling activator) (e). Statistically significant differences compared to Col-0 were determined by ordinary one-way ANOVA followed by Tukey's multiple comparison test, two-sided. Box plots display 2nd and 3rd quartiles and the median, whiskers indicate maximum and minimum.

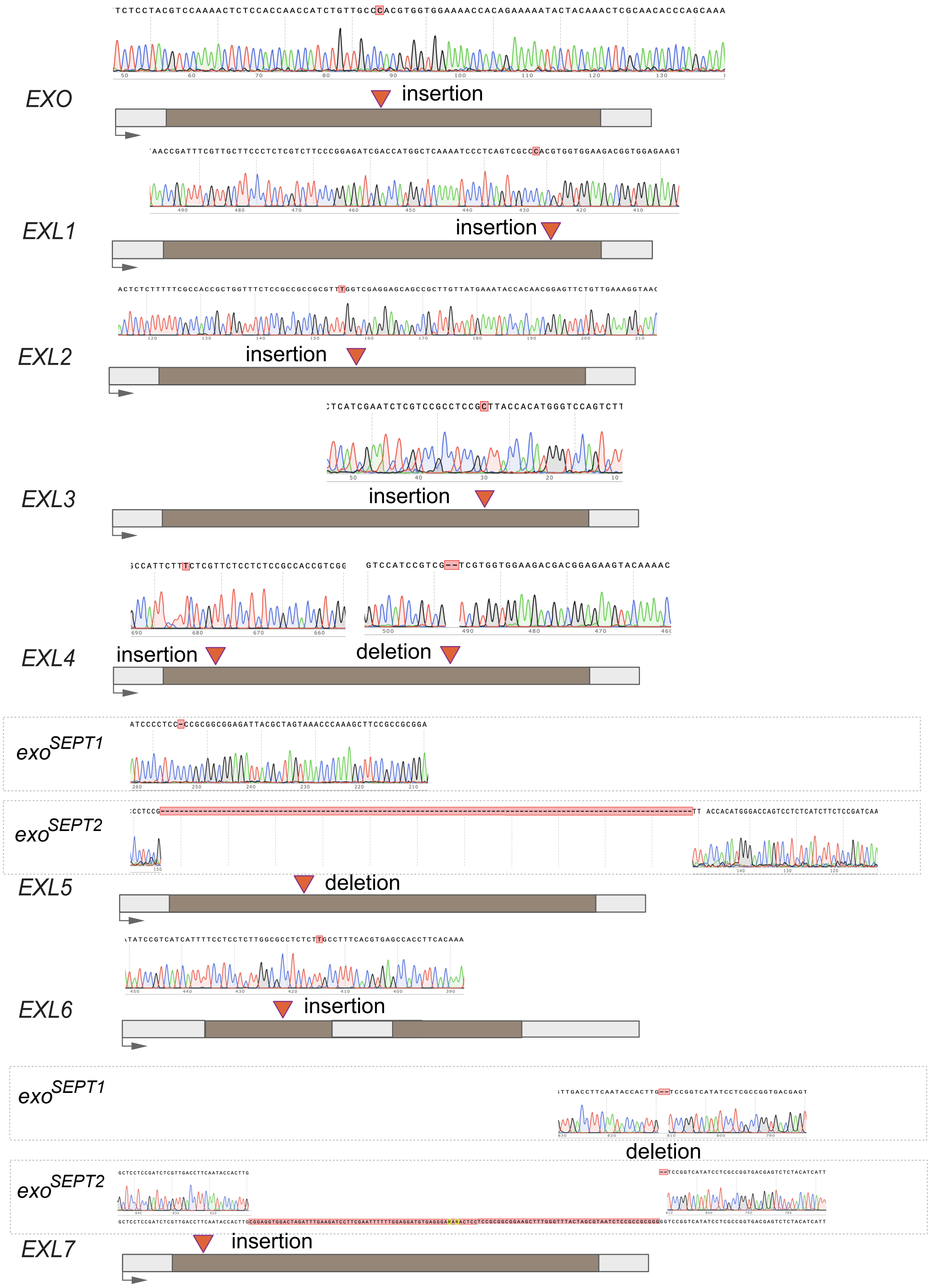

Supporting Figure S7: CRISPR/Cas9-generated mutants in *EXO/EXL* genes. Schematic representation of *EXO/EXL* genes for which loss-of-function mutants were obtained. The open reading frames are shaded in brown. Sequence traces of the CRISPR/Cas9-generated homozygous alleles are shown above each gene schematic.

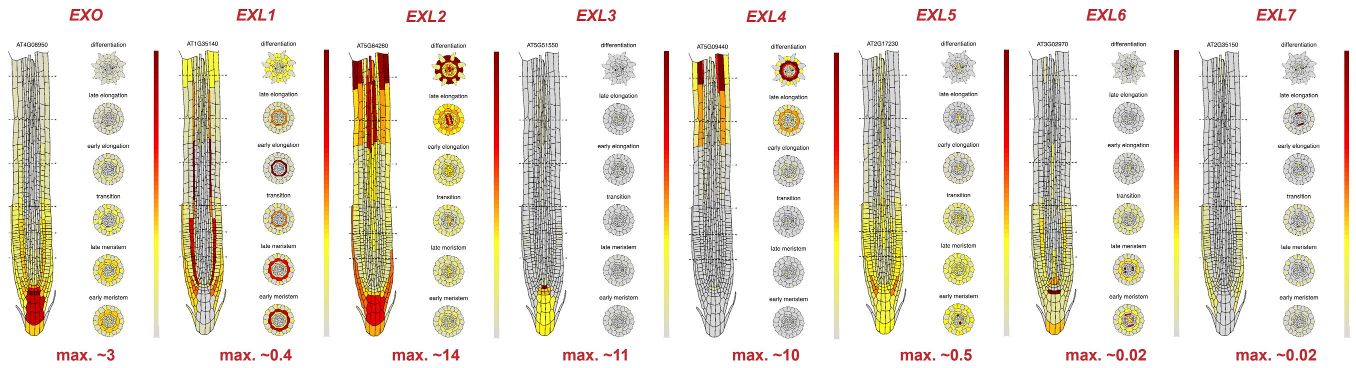

Supporting Figure S8: Expression patterns of *EXO/EXL* genes. Schematic representation of root tip expression patterns of *EXO/EXL* genes, obtained from aggregation of multiple independent scRNAseq experiments of Arabidopsis Col-0 wildtype roots (https://rootcellatlas.org). Note the differences in expression level scales.

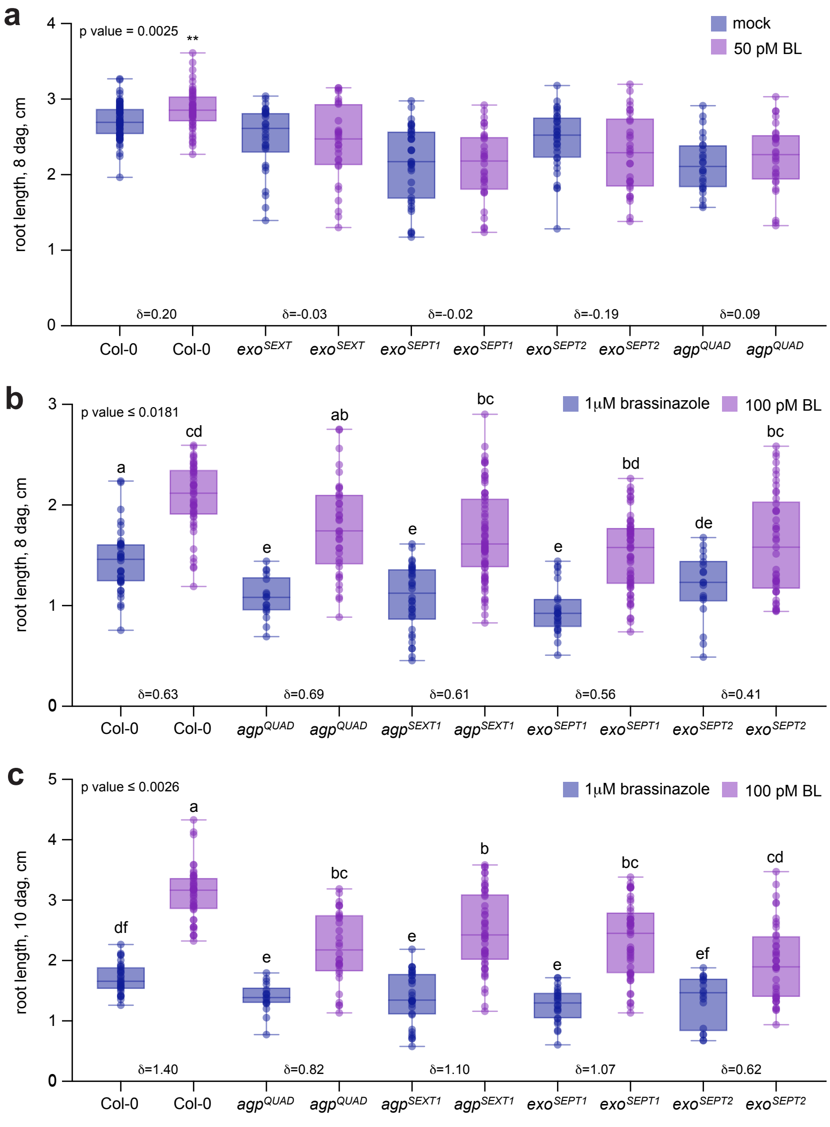

Supporting Figure S9: Brassinosteroid response of *agp* and *exo/exl* mutants. (a) Root length measurements of seedlings from indicated genotypes and treatments. Statistically significant differences (asterisks) compared to mock treatment were determined by ordinary one-way ANOVA followed by Welch's t-test, two-sided. **(b-c)** Root length measurements of seedlings from indicated genotypes, either continuously grown on brassinazole or transferred onto brassinolide after 3 days. Statistically significant differences (letters; p≦0.05) were determined by Brown-Forsythe and Welch ANOVA test, two-sided. d: differences (cm) between averages of treatments and controls. Box plots display 2nd and 3rd quartiles and the median, whiskers indicate maximum and minimum. **: p≦0.01;

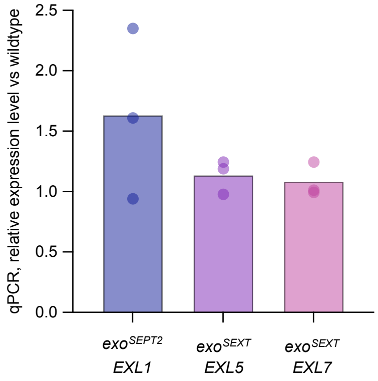

Supporting Figure S10: Expression levels of *EXL* genes. qPCR expression level measurements of remaining intact *EXL* genes in the sextuple or septuple *exo/exl* mutant backgrounds as compared to wildtype. The *SAND* gene (At2g28390) was used as a reference.
